# Energy drink consumption and associated lifestyle factors among German adolescents: findings from the EDKAR-study

**DOI:** 10.1007/s00394-026-04153-x

**Published:** 2026-11-11

**Authors:** Maria J. Pie, Sven Knüppel, Britta Nagl, Anke Ehlers, Fabian Spinka, Andrea Deichl, Frank Edelmann, Cornelia Weikert, Juliane Menzel

**Affiliations:** 1 Department Food and Feed Safety in the Food Chain, German Federal Institute for Risk Assessment, Max-Dohrn-Straße 8-10, 10589 Berlin, Germany; 2 Institute of Social Medicine, Epidemiology and Health Economics, Charité – University Medical Center Berlin, Corporate Member of Freie Universität Berlin and Humboldt- Universität zu Berlin, Berlin, Germany; 3 German Center for Cardiovascular Research (DZHK), Partner Site Berlin, Berlin, Germany; 4 Department of Cardiology, Angiology and Intensive Care Medicine, Deutsches Herzzentrum der Charité (DHZC) – Medical Heart Center of Charité and German Heart Institute Berlin, Berlin, Germany; 5 Charité – University Medical Center Berlin, Corporate Member of Freie Universität Berlin and Humboldt-Universität zu Berlin, Berlin, Germany

**Keywords:** Energy drink, Adolescents, Lifestyle, Substance use, Sleep, Caffeinated beverages

## Abstract

**Purpose:**

The consumption of energy drinks (EDs) has become increasingly popular among adolescents. The present study aimed to comprehensively characterize ED consumption behavior and associated lifestyle factors in pupils aged 15–18 years.

**Methods:**

The cross-sectional EDKAR-study assessed data from 5100 German pupils using an online questionnaire. Following exclusion criteria, 4893 pupils were categorized into six ED consumer groups based on the frequency, duration, and quantity of their reported ED consumption: non-consumers, occasional, moderate, regular, chronic low, and chronic high consumers.

**Results:**

Overall, 65.6% (*n* = 3208) reported ED consumption within the past 12 months, 12.6% on ≥ 4 days per week, and 3.9% daily. Median (IQR) daily ED intake was higher in groups with more frequent ED consumption, ranging from 20.5 ml/day (5.3–41.0) among occasional consumers to 818 ml/day (571–1250) among chronic high consumers. Reported adverse effects likewise increased from occasional to chronic high consumers, ranging from 23.2% to 50.2%, with palpitations, poor sleep, headaches, and chest pressure reported most frequently. Harmful lifestyle behaviors also increased from non-consumers to chronic high consumers, including alcohol intake (from 0.2 to 2.3 glasses per week), smoking rates (from 5.6% to 53.3%; cannabis: from 10.3% to 48.8%), short sleep duration (< 5 h/night on weekdays: from 4.7% to 20.6%), and excessive daily screen time (> 8 h/day: from 35.8% to 72.8%).

**Conclusion:**

The EDKAR-study highlights the high prevalence of ED consumption among German adolescents and its association with several harmful lifestyle behaviors, underscoring the importance of raising awareness of potential adverse health effects of ED consumption.

**Supplementary Information:**

The online version contains supplementary material available at https://doi.org/10.1007/s00394-026-04153-x.

## Introduction

Energy drinks (EDs) are highly caffeinated beverages that contain next to caffeine one or more additional ingredients such as taurine, glucuronolactone, or inositol, and are typically sweetened with either sugar or artificial sweeteners [1]. They are commonly marketed as products that boost energy levels, enhance mental alertness, and increase physical or cognitive performance [1, 2].

In recent years, EDs have become increasingly popular and remain widely consumed among adolescents and young adults [1, 3–5]. In fact, a recently published survey in Germany from 2024 (*n* = 1000, aged 14 to 18 years) noticed that 57% of adolescents reported consuming EDs, with 19% reporting ED consumption several times a month, 8% several times a week, and 2% even daily [6]. Similarly, one of the most comprehensive multinational surveys conducted by the European Food Safety Authority (EFSA) in 2013 across 16 European countries (*n* = 31070, aged 10 to 18 years) reported consistently high rates of ED consumption within the past year [3]. Accordingly, the average prevalence of ED consumption among adolescents is approximately 68%, with national rates ranging from 48% in Greece to 85% in Belgium [3]. In Germany, a prevalence rate of 60% for ED consumption has been reported [3]. Of these German ED consumers, about 9% were classified as chronic high consumers, consuming EDs at least four to five times per week with an average intake of 7 L per month [3]. In this context, the EFSA derived a maximum daily caffeine intake of 3 mg per kilogram of body weight (BW) as a safe limit for children and adolescents [7]. For a 50 kg adolescent, this corresponds to 150 mg of caffeine and would already be exceeded by consuming two standard 250 ml ED cans containing 32 mg of caffeine per 100 ml.

Previous studies noticed that ED consumption is associated with a broader range of harmful lifestyle behaviors, including the co-consumption of EDs with other substances such as alcohol, tobacco, and cannabis [5, 8, 9]. Of particular concern is the mixing of EDs with alcohol, which has been shown to diminish the perception of alcohol intoxication and thereby increase the risk of alcohol-related injuries and engagement in risky behaviors [8, 10]. Additionally, poor sleep hygiene and increased screen time have been identified as further lifestyle behaviors associated with ED consumption [9, 11].

Given the widespread consumption of EDs among adolescents, along with previous study findings linking their consumption to harmful lifestyle behaviors as well as adverse health effects (e.g., chest pain, sleep problems, and anxiety), significant concerns have been raised regarding their safety [1, 2, 5, 12]. Due to the lack of studies on ED consumption among minors, especially in Germany, the present EDKAR-study aimed to comprehensively investigate the consumption of EDs among pupils aged 15 to 18 years in Berlin, Germany [13]. In more detail, the study aimed to provide a descriptive characterization of six predefined ED consumer groups, i.e., non-consumers of EDs, occasional ED consumers, moderate ED consumers, regular ED consumers, chronic low ED consumers, and chronic high ED consumers. This included various aspects of ED consumption, such as frequency, duration, and quantity of reported ED intake, caffeine intake from EDs and other caffeinated beverages, mixed consumption of EDs with alcohol, and reported adverse effects following ED consumption. Furthermore, the study aimed to describe sociodemographic and lifestyle characteristics across these consumer groups, particularly physical activity, cigarette and cannabis smoking, alcohol consumption, sleep behavior, and media use.

## Methods

### Study design

The study on chronic high ED consumption and cardiovascular risk (EDKAR-study) is a cross-sectional study conducted in Berlin (Germany), including adolescents aged 15 to 18 years. The EDKAR-study was conducted in two phases between March 2022 and May 2024. In the first study phase, a comprehensive online questionnaire was used to collect data on ED consumption (sample size: *n* = 5000 pupils) with the aim of identifying adolescents with chronically high ED consumption and establishing a control group for the second phase of the study (cardiological examinations), as described elsewhere [13].

Following approval from the Berlin Senate Department for Education, Youth and Family, pupils were recruited. Over the entire project period, a total of 156 school principals (from grammar schools, secondary schools, and vocational high school centers) across Berlin were contacted by telephone and/or email and asked to participate with suitable classes (aged 15–18 years) in study phase 1. Once the school principals had agreed, the study team presented the EDKAR-study to the pupils in person in the classroom, showed a short explanatory video, handed out written study information to all pupils, and answered open questions regarding study participation. Each pupil also received a written study information for their parents or legal guardians. All participants were fully informed about the EDKAR-study and informed consent was obtained from each pupil before they received personalized access to the online questionnaire. Study phase 1 of the EDKAR-study complied with the official requirements of the Berlin Senate Department for Education, Youth and Family, according to which an informed consent from pupils aged 14 years and older is required, while parents or guardians had to be provided with written study information. Given that this regulation applies to Berlin, only Berlin schools were considered in study phase 1. In total, 29 Berlin schools with 359 classes supported the EDKAR-study and were included in study phase 1.

The study procedures were approved by the Ethics Committee of Charité University Medical Center Berlin (no. EA2/176/21) and were conducted in accordance with the Declaration of Helsinki. The study was registered in the German Clinical Trials Register under DRKS00026698 (registered 15 November 2021).

### Data collection

For the EDKAR-study, a comprehensive online questionnaire was developed to assess various domains, including ED consumption and the consumption of other caffeinated beverages, as well as sociodemographic data, leisure activities, media use, and health-related behaviors such as sleep behavior, smoking behavior, and alcohol consumption.

#### Assessment of energy drink consumption

The questionnaire assessed variables related to ED consumption, including lifetime ED consumption (yes, tried once with less than one can, or no), ED consumption within the past 12 months (yes or no), frequency of ED consumption within the past 12 months (from “once per month or less” to “daily”), and duration of ED consumption (from “less than 6 months” to “more than 2 years”). To assess the quantity of EDs consumed on a consumption day, participants were asked to indicate the number of EDs consumed for each predefined can or bottle size, ranging from 250 ml to 1.5 L, with response options ranging from 0 to 10 for each portion size. Participants also reported their usual preference for sugar-containing or sugar-free EDs, how often they consumed EDs and alcohol together (from “never” to “always”), and their main motives for consuming EDs. Participants were also asked to report whether they had experienced any adverse effects following ED consumption (yes or no); if yes, the adverse effects were specified using multiple predefined response options, including a free-text response option. Usual daily ED intake (ml/day) was calculated based on the reported frequency and quantity of ED consumption. Three categories of inconsistent responses were determined: (1) “don’t know/no answer”, representing missing information, (2) “inconsistent zero intake”, defined as a calculated intake of 0.0 L on a consumption day, which was inconsistent with reported ED consumption in general, and (3) “implausibly high ED intake”, defined as a calculated ED intake exceeding 5.0 L on a single consumption day, which was considered physiologically implausible. All three categories were excluded from intake-based calculations. Based on the usual daily ED intake and a typical ED caffeine content of 32 mg/100 ml, usual daily caffeine intake from EDs (mg/day) and weight-adjusted caffeine intake from EDs (mg/kg BW/day) were calculated.

Participants were classified into consumption groups based on their ED consumption behavior. Non-consumers were defined as adolescents who either never consumed EDs (*n* = 643), tried them only once in their lifetime (less than one can) (*n* = 734), or former ED consumers who had not consumed EDs within the past 12 months (*n* = 308). ED consumers were further categorized into five consumption groups based on three dimensions of reported ED consumption: consumption frequency within the past 12 months, duration of consumption, and daily weight-adjusted caffeine intake from EDs. Occasional ED consumers reported consuming EDs one to three times per month. Moderate ED consumers reported consuming EDs one to three times per week. Regular ED consumers reported consuming EDs at least four times per week for less than one year, with unrestricted weight-adjusted caffeine intake from EDs (mg/kg BW/day). Chronic low ED consumers reported consuming EDs at least four times per week for at least one year while remaining below the EFSA-recommended safe upper intake limit for caffeine of 3 mg/kg BW/day [7]. Chronic high ED consumers reported consuming EDs at least four times per week for at least one year while exceeding the daily caffeine threshold of 3 mg/kg BW/day through ED consumption alone.

#### Assessment of caffeine-containing beverages

Moreover, the questionnaire assessed caffeine-containing beverages, including coffee drinks, black and green tea, chocolate drinks, cola or mate beverages, iced tea, and pre-workout boosters. For each beverage category selected, participants reported how often they had consumed it within the past 12 months (from “never” to “daily”) and how much they usually consumed it per occasion (from “half a serving” to “four servings or more”). The usual daily caffeine intake (mg/day) and weight-adjusted daily caffeine intake (mg/kg BW/day) from all other caffeine-containing beverages (excluding EDs) were calculated based on the reported consumption frequency and quantity of coffee drinks (90 mg of caffeine per cup), black or green tea (38 mg of caffeine per cup), drinking chocolate (30 mg of caffeine per cup), cola or mate soft drinks (31 mg caffeine per glass), iced tea (10 mg of caffeine per glass), and pre-workout or training boosters (225 mg of caffeine per serving). Additionally, the usual daily caffeine intake (mg/day) and usual weight-adjusted daily caffeine intake (mg/kg BW/day) from all caffeine-containing beverages including EDs were calculated.

#### Assessment of sociodemographic and lifestyle characteristics

The questionnaire assessed sociodemographic variables, including gender (male, female, or diverse), age (15, 16, 17, or 18 years), school type (grammar school, secondary school, or vocational school center), country of birth (Germany or other country), as well as self-reported height and body weight entered either as free text or by selecting predefined categories. The formula for body mass index (BMI) comprises weight in kilograms divided by height in meters squared. BMI calculations were based either on self-reported weight and height values or on middle values derived from predefined weight and height categories. Due to missing in weight and height, values were estimated using a regression model (*n* = 4 each for weight and height). BMI percentile categories were defined according to the German reference values for adolescents as underweight (< P10), normal weight (P10–P90), overweight (> P90–P97), and obesity (> P97) [14].

Harmful lifestyle behaviors included smoking status, smoking cannabis (herb and resin), alcohol consumption, and sleep behavior. Smoking status was categorized as smoker, ex-smoker, or non-smoker and included the use of conventional cigarettes, e-cigarettes, e-shisha, shisha, and tobacco heaters. Smoking cannabis included lifetime cannabis use (yes or no) and cannabis consumption frequency within the past 12 months (from “once per month or less” to “daily”). Alcohol consumption included lifetime alcohol consumption (yes or no), frequency of alcohol consumption within the past 12 months (from “once per month or less” to “daily”), and the usual quantity of alcohol consumed per occasion (from “half a glass” to “four glasses or more”). Glasses of alcohol per week were calculated based on the reported frequency and quantity of alcoholic beverages. Sleep behavior included usual sleep duration on weekdays and weekends (from “<5 h” to “>11 h”) and sleep disturbances (“Yes, I have difficulty falling asleep”, “Yes, I have difficulty sleeping through the night”, or “No”).

The assessment of leisure activities included the frequency of physical activity, meeting friends outside of school hours, video calling with friends or other people, and gaming activities such as playing PlayStation, Xbox, Nintendo, or computer games (from “never” to “daily”). The daily duration of media use included gaming on consoles, computers, tablets, or smartphones, the use of messenger services, and the use of social networks (from “not at all” to “more than 3 hours” per day). Additional leisure activity and media use variables are provided in the Supplementary Material. To calculate usual daily screen time (hours/day), the following predefined response categories were converted into estimated screen time durations: “not at all” = 0 h, “up to 1 hour” = 0.5 h, “up to 3 hours” = 2 h, and “more than 3 hours” = 4 h. Total daily screen time was calculated as the sum of the following activity categories: watching online videos or using streaming platforms, watching television or DVDs, gaming on consoles, computers, tablets, or smartphones, using messenger services, using social media platforms on mobile devices, and general internet browsing. Additionally, adolescents were categorized into screen time groups of “up to 5 hours”, “5 to 8 hours”, and “more than 8 hours” per day.

### Final study population

**Fig. 1:**
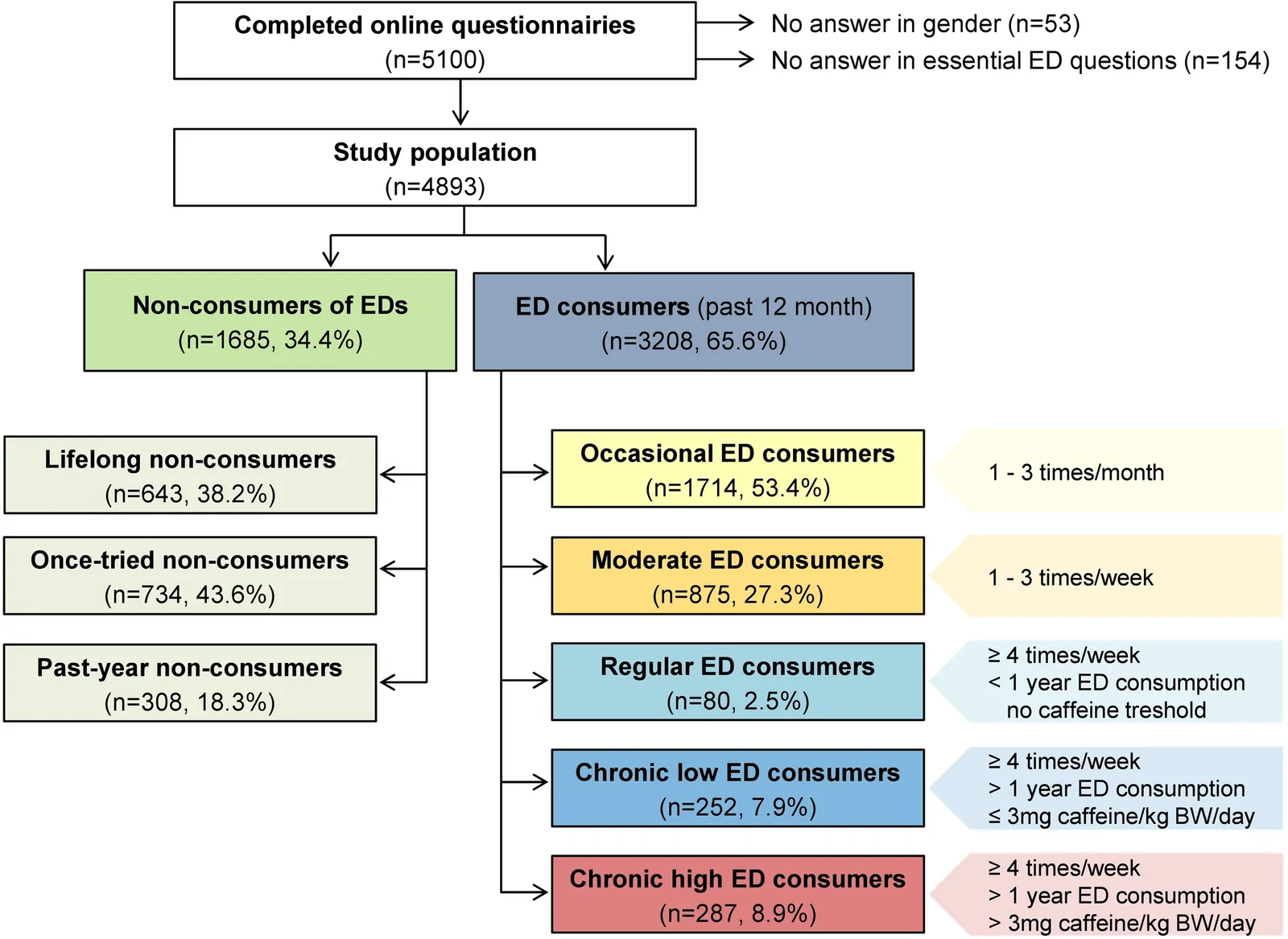
Flow chart illustrating the EDKAR-study (study phase 1) and the categorization of ED consumer groups based on ED consumption within the past 12 months. The non-consumer group comprises adolescents who had never consumed EDs, those who had tried EDs only once (less than one can), and former consumers who had not consumed EDs within the previous 12 months

A total of 5100 participants fully completed the online questionnaire. However, 207 adolescents had to be excluded due to the “don’t know/no answer” responses regarding gender (*n* = 53) or ED consumption frequency and duration (*n* = 154). The final study population therefore comprised 4893 adolescents (Fig. 1). For all other variables, “don’t know/no answer” responses are presented in the tables. The power calculation has been described elsewhere [13].

### Statistical analysis

The data from study phase 1 were used for a comprehensive descriptive characterization of different ED consumption groups regarding ED consumption behavior as well as various sociodemographic and lifestyle characteristics. Categorical variables were reported as percentages with corresponding absolute frequencies. For selected key findings, 95% confidence intervals (95% CI) were calculated and reported in the text. Normality of continuous variables was assessed by visual inspection of histograms and normal probability plots, as well as by evaluating skewness and kurtosis. Normally distributed continuous variables were reported as means with standard deviations (SD), otherwise as medians with interquartile ranges (IQR). Quintiles were calculated separately for usual daily ED intake (mL/day), usual daily caffeine intake from EDs (mg/day), and usual daily weight adjusted caffeine intake from EDs (mg/kg BW/day). All statistical analyses were performed using SAS software, version 9.4 (SAS Institute Inc., Cary, NC, USA).

## Results

The final study population comprised 4893 adolescents (mean ± SD age: 16.2 ± 1.0 years; 53.8% male) from grammar schools (29.6%), secondary schools (47.3%), and vocational school centers (23.1%). Among them, 20.3% reported smoking, 25.7% had smoked cannabis (herb and resin), and 67.3% had consumed alcohol (Table 1).

### Energy drink consumption characteristics

Overall, 71.9% (*n* = 3516) of participants had ever consumed EDs in their lifetime, 15.0% (*n* = 734) had tried EDs only once with less than one can, and 13.1% (*n* = 643) had never consumed EDs. Former ED consumers who had not consumed EDs within the previous 12 months showed only minor differences in baseline characteristics compared with lifelong non-consumers and tried-once non-consumers (Supplementary Table S1). These three subgroups were therefore combined into a single non-consumer group. Accordingly, 65.6% (*n* = 3208) of all adolescents were classified as ED consumers within the past 12 months, with 12.6% reporting ED consumption on at least four days per week and 3.9% reporting daily consumption (Supplementary Figure S1). Regarding ED consumption duration, 22.2% (*n* = 714) of ED consumers reported consuming EDs for less than one year, 17.3% (*n* = 555) for one to two years, and 56.0% (*n* = 1796) for more than two years (Supplementary Figure S2). Usual daily weight-adjusted caffeine intake from EDs ranged from a median of 1.1 mg/kg BW/day (IQR: 0.9–1.1) in the lowest quintile to 7.0 mg/kg BW/day (IQR: 5.3–10.5) in the highest quintile (Supplementary Table S2). Based on these three dimensions of their ED consumption, all participants were classified into the ED consumption groups: 34.4% (*n* = 1685) non-consumers of EDs, 35.0% (*n* = 1714) occasional ED consumers, 17.9% (*n* = 875) moderate ED consumers, 1.6% (*n* = 80) regular ED consumers, 5.2% (*n* = 252) chronic low ED consumers and 5.9% (*n* = 287) chronic high ED consumers.

The median usual daily ED intake among all ED consumers was 82.9 ml/day (IQR: 20.5–286) (Table 2). Across the consumption groups the usual daily intake of EDs intake increased, ranging from a median of 20.5 ml/day (IQR: 5.3–41.0) among occasional consumers to 818 ml/day (IQR: 571–1250) in chronic high ED consumers (Table 2). Caffeine intake from EDs alone was in median 26.5 mg/day (IQR: 6.6–91.4) among all ED consumers and ranged from 6.6 mg/day (IQR: 1.7–13.1) among occasional consumers to 262 mg/day (IQR: 183–400) in chronic high ED consumers, corresponding to 4.9 mg/kg BW/day (IQR: 3.9–7.6) in the latter group. Caffeine intake from other caffeinated beverages also increased with higher ED consumption (Table 3). Regarding high ED intake of more than one liter on a single consumption day, this was not only reported by 44.9% (*n* = 129) of chronic high ED consumers, but also by 14.2% (*n* = 124) of moderate ED consumers and 17.5% (*n* = 14) of regular ED consumers (Table 2). The preference for sugar-sweetened EDs increased across the consumption groups, with the highest proportions observed in chronic low (62.7%) and chronic high ED consumers (64.5%) (Table 2). Likewise, mixing EDs with alcohol increased across the ED consumption groups and was most frequent among chronic high ED consumers (Table 2).

**Fig. 2:**
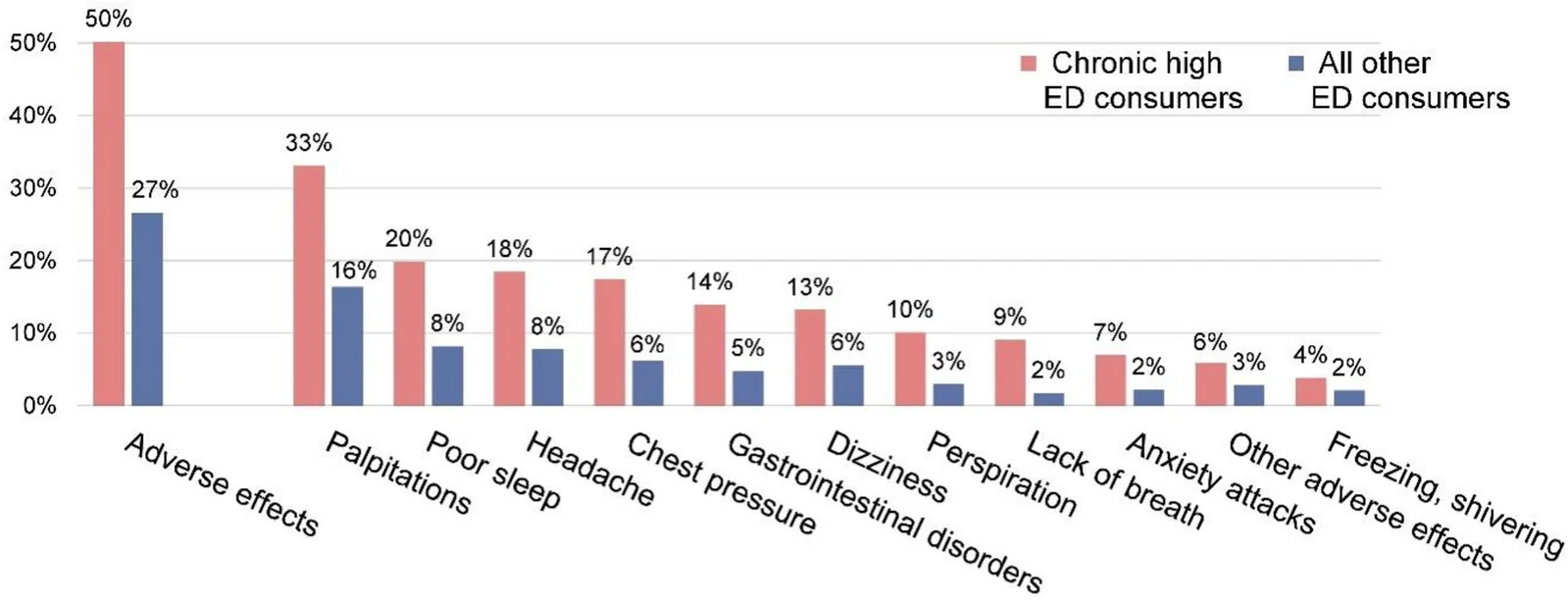
Adverse effects experienced after ED consumption among chronic high ED consumers (*n* = 287), compared with all other ED consumers (*n* = 2921). All other ED consumers include the consumption groups occasional, moderate, regular, and chronic low ED consumers. Multiple answers were allowed for the question on adverse effects

Adverse effects following ED consumption were reported by 28.7% of all consumers, rising from 23.2% among occasional consumers to 50.2% among chronic high ED consumers (Table 2). Across all ED consumption groups, the most frequently reported adverse effects were consistently palpitations, poor sleep, headaches, and a feeling of chest pressure (Table 2). However, the frequency of all reported adverse effects increased with higher ED consumption, with chronic high ED consumers consistently reporting the highest percentages across all symptoms (Fig. 2; Table 2). The main reasons for consuming EDs were taste (68.0%), waking up (45.6%), and staying awake longer (35.3%) (Supplementary Table S3).

**Table 1:** Study characteristics of the study population (EDKAR-study phase 1) stratified by ED consumption groups

	Overall population (*n* = 4893)	Non-consumers of EDs (*n* = 1685)	Occasional ED consumers (*n* = 1714)	Moderate ED consumers (*n* = 875)	Regular ED consumers (*n* = 80)	Chronic low ED consumers (*n* = 252)	Chronic high ED consumers (*n* = 287)
*Gender*							
Male	53.8% (2633)	52.2% (880)	53.4% (915)	57.4% (502)	61.3% (49)	54.4% (137)	52.3% (150)
Female	44.8% (2193)	46.9% (790)	45.3% (776)	41.1% (360)	38.8% (31)	42.9% (108)	44.6% (128)
Diverse	1.4% (67)	0.9% (15)	1.3% (23)	1.5% (13)	–	2.8% (7)	3.1% (9)
Age [years]	16.2 ± 1.0	16.0 ± 1.0	16.2 ± 1.0	16.2 ± 1.0	16.4 ± 1.0	16.5 ± 1.0	16.2 ± 1.0
BMI [kg/m^2^]	21.5 (19.4–24.1)	20.8 (19.0–23.2)	21.5 (19.5–24.0)	22.4 (20.2–25.2)	22.4 (19.9–24.6)	22.5 (20.4–25.7)	21.6 (19.5–24.6)
*School type*							
Grammar school	29.6% (1449)	38.2% (644)	31.6% (542)	19.5% (171)	8.8% (7)	18.3% (46)	13.6% (39)
Secondary school	47.3% (2315)	43.8% (738)	47.1% (808)	51.4% (450)	57.5% (46)	50.8% (128)	50.5% (145)
Vocational school center	23.1% (1129)	18.0% (303)	21.2% (364)	29.0% (254)	33.8% (27)	31.0% (78)	35.9% (103)
*Country of birth*							
Germany	86.9% (4251)	86.3% (1454)	87.7% (1503)	85.4% (747)	90.0% (72)	90.9% (229)	85.7% (246)
Other country	12.9% (630)	13.5% (227)	12.0% (206)	14.6% (128)	10.0% (8)	9.1% (23)	13.2% (38)
Don’t know/no answer	0.2% (12)	0.2% (4)	0.3% (5)	–	–	–	1.0% (3)
*Physical activity*							
Never	9.2% (449)	8.6% (145)	9.1% (156)	7.7% (67)	12.5% (10)	13.1% (33)	13.2% (38)
Less than once a week	9.8% (478)	9.1% (154)	9.2% (157)	10.6% (93)	11.3% (9)	10.7% (27)	13.2% (38)
Once a week	18.0% (881)	17.6% (296)	19.8% (339)	16.5% (144)	8.8% (7)	15.1% (38)	19.9% (57)
2–6 times a week	49.7% (2432)	50.3% (848)	50.0% (857)	51.3% (449)	50.0% (40)	46.4% (117)	42.2% (121)
Daily	11.1% (541)	12.0% (203)	10.3% (177)	10.7% (94)	13.8% (11)	10.7% (27)	10.1% (29)
Don’t know/no answer	2.3% (112)	2.3% (39)	1.6% (28)	3.2% (28)	3.8% (3)	4.0% (10)	1.4% (4)
*Smoking status* ^a^							
Smoker	20.3% (993)	5.6% (94)	17.6% (301)	33.0% (289)	37.5% (30)	50.0% (126)	53.3% (153)
Ex-smoker	13.3% (653)	6.8% (115)	16.1% (276)	18.1% (158)	17.5% (14)	14.7% (37)	18.5% (53)
Non-smoker	63.5% (3107)	85.8% (1445)	63.0% (1079)	45.5% (398)	40.0% (32)	32.5% (82)	24.7% (71)
Don’t know/no answer	2.9% (140)	1.8% (31)	3.4% (58)	3.4% (30)	5.0% (4)	2.8% (7)	3.5% (10)
*Smoking cannabis (herb and resin)*							
Yes	25.7% (1258)	10.3% (174)	27.4% (469)	36.2% (317)	40.0% (32)	50.0% (126)	48.8% (140)
No	72.3% (3538)	88.1% (1485)	70.8% (1213)	61.1% (535)	58.8% (47)	48.0% (121)	47.7% (137)
Don’t know/no answer	2.0% (97)	1.5% (26)	1.9% (32)	2.6% (23)	1.3% (1)	2.0% (5)	3.5% (10)
If yes, daily cannabis smokers	1.1% (53)	0.2% (3)	0.5% (9)	1.7% (15)	1.3% (1)	2.0% (5)	7.0% (20)
*Alcohol consumption*							
Yes	67.3% (3292)	54.5% (918)	72.5% (1243)	74.9% (655)	70.0% (56)	77.8% (196)	78.0% (224)
No	31.8% (1555)	44.2% (744)	27.0% (463)	24.0% (210)	30.0% (24)	21.8% (55)	20.6% (59)
Don’t know/no answer	0.9% (46)	1.4% (23)	0.5% (8)	1.1% (10)	–	0.4% (1)	1.4% (4)
If yes, Glasses of alcohol per week ^b^	0.6 (0.1–2.3)	0.2 (0.1–1.7)	0.5 (0.1–2.3)	1.1 (0.2–3.0)	1.1 (0.5–4.0)	2.0 (0.5–4.0)	2.3 (0.5–8.0)
If yes, ≥ 4 glasses on one occasion	17.4% (852)	7.8% (132)	17.6% (302)	23.0% (201)	33.8% (27)	33.3% (84)	36.9% (106)
*Sleep disturbances*							
Yes	34.6% (1694)	28.7% (484)	32.1% (550)	39.7% (347)	42.5% (34)	44.4% (112)	58.2% (167)
No	59.9% (2933)	65.2% (1098)	62.4% (1070)	56.0% (490)	50.0% (40)	51.2% (129)	36.9% (106)
Don’t know/no answer	5.4% (266)	6.1% (103)	5.5% (94)	4.3% (38)	7.5% (6)	4.4% (11)	4.9% (14)
If yes, difficulty falling asleep ^c^	76.0% (1288)	77.7% (376)	73.8% (406)	76.7% (266)	79.4% (27)	74.1% (83)	77.8% (130)
If yes, difficulty staying asleep ^b^	46.9% (795)	40.1% (194)	45.5% (250)	50.7% (176)	44.1% (15)	59.8% (67)	55.7% (93)

Variables expressed as percentage (n), mean (± SD), or median (IQR).

^a^ Smoking included consumption of cigarettes, shisha, e-cigarettes, e-shisha, or tobacco heaters.

^b^ Missings: *n* = 452.

^c^ Multiple answers possible.

**Table 2:** Various aspects of ED consumption behavior stratified by ED consumption groups

	Overall ED consumers (*n* = 3208)	Occasional ED consumers (*n* = 1714)	Moderate ED consumers (*n* = 875)	Regular ED consumers (*n* = 80)	Chronic low ED consumers (*n* = 252)	Chronic high ED consumers (*n* = 287)
*ED intake on a consumption day*						
Up to 0.5 L	45.9% (1471)	41.2% (706)	57.0% (499)	40.0% (32)	84.9% (214)	7.0% (20)
Up to 1.0 L	15.3% (490)	8.6% (147)	19.5% (171)	30.0% (24)	12.3% (31)	40.8% (117)
More than 1.0 L	11.6% (371)	6.0% (103)	14.2% (124)	17.5% (14)	0.4% (1)	44.9% (129)
Don’t know/no answer ^a^	0.6% (19)	0.7% (12)	0.5% (4)	3.8% (3)	–	–
Inconsistent zero intake (0.0 L) ^a^	25.1% (805)	43.0% (737)	6.7% (59)	3.8% (3)	2.4% (6)	–
Implausible high intake (> 5.0 L) ^a^	1.6% (52)	0.5% (9)	2.1% (18)	5.0% (4)	–	7.3% (21)
*Usual daily ED consumption*						
Inconsistent answers ^a^	27.3% (876)	44.2% (758)	9.3% (81)	12.5% (10)	2.4% (6)	7.3% (21)
Usual daily intake of EDs [ml/day]	82.9 (20.5–286)	20.5 (5.3–41.0)	107 (71.5–215)	428 (286–786)	286 (188–393)	818 (571–1250)
Usual daily caffeine intake from EDs [mg/day]	26.5 (6.6–91.4)	6.6 (1.7–13.1)	34.3 (22.9–68.6)	137 (91.4–252)	91.4 (60.3–126)	262 (183–400)
Usual daily weight-adjusted caffeine intake from EDs [mg/kg BW/day]	2.0 (1.3–3.7)	1.6 (1.2–2.8)	2.0 (1.3–3.5)	3.0 (1.8–4.9)	1.8 (1.3–2.5)	4.9 (3.9–7.6)
*ED with/without sugar*						
With sugar	52.2% (1674)	47.8% (820)	52.9% (463)	60.0% (48)	62.7% (158)	64.5% (185)
Without sugar (e.g., “zero”, “sugar-free”)	12.7% (407)	13.1% (224)	14.3% (125)	6.3% (5)	12.3% (31)	7.7% (22)
Half and half	29.7% (952)	30.1% (516)	31.9% (279)	26.3% (21)	23.8% (60)	26.5% (76)
Don’t know/no answer	5.5% (175)	9.0% (154)	0.9% (8)	7.5% (6)	1.2% (3)	1.4% (4)
*ED mixed with alcohol*						
Never	58.4% (1875)	66.2% (1134)	54.2% (474)	52.5% (42)	44.4% (112)	39.4% (113)
Rarely	20.3% (650)	19.1% (328)	22.2% (194)	18.8% (15)	23.8% (60)	18.5% (53)
Sometimes	13.6% (436)	9.6% (165)	14.5% (127)	18.8% (15)	20.2% (51)	27.2% (78)
Often	5.4% (172)	2.9% (49)	6.9% (60)	10.0% (8)	9.1% (23)	11.1% (32)
Always	1.5% (49)	1.5% (25)	1.3% (11)	–	0.8% (2)	3.8% (11)
Don’t know/no answer	0.8% (26)	0.8% (13)	1.0% (9)	–	1.6% (4)	–
*Adverse effects*						
Yes	28.7% (922)	23.2% (398)	29.3% (256)	36.3% (29)	37.7% (95)	50.2% (144)
No	66.1% (2122)	70.9% (1215)	66.1% (578)	58.8% (47)	59.1% (149)	46.3% (133)
Don’t know/no answer	5.1% (164)	5.9% (101)	4.7% (41)	5.0% (4)	3.2% (8)	3.5% (10)
*Reported adverse effects (multiple answers)*						
Palpitations	17.9% (574)	13.5% (232)	18.2% (159)	18.8% (15)	29.0% (73)	33.1% (95)
Poor sleep	9.3% (297)	7.1% (121)	9.3% (81)	11.3% (9)	11.5% (29)	19.9% (57)
Headache	8.8% (281)	7.1% (122)	7.7% (67)	7.5% (6)	13.1% (33)	18.5% (53)
Chest pressure	7.2% (231)	4.8% (82)	6.5% (57)	13.8% (11)	12.3% (31)	17.4% (50)
Dizziness	6.3% (201)	5.1% (88)	4.6% (40)	10.0% (8)	10.7% (27)	13.2% (38)
Gastrointestinal disorders	5.6% (181)	4.3% (74)	5.3% (46)	13.8% (11)	4.0% (10)	13.9% (40)
Perspiration	3.6% (116)	2.4% (41)	3.2% (28)	8.8% (7)	4.4% (11)	10.1% (29)
Other adverse effects	3.1% (101)	2.0% (34)	4.6% (40)	–	4.0% (10)	5.9% (17)
Anxiety attacks	2.6% (85)	2.1% (36)	1.7% (15)	2.5% (2)	4.8% (12)	7.0% (20)
Lack of breath	2.4% (76)	1.8% (30)	1.0% (9)	1.3% (1)	4.0% (10)	9.1% (26)
Freezing, shivering	2.3% (73)	2.0% (35)	2.3% (20)	–	2.8% (7)	3.8% (11)
Don’t know/no answer	0.4% (12)	0.4% (6)	0.2% (2)	–	–	1.4% (4)

Variables expressed as percentage (n) or median (IQR)

^a^ Inconsistent answers included “don’t know/no answer” (*n* = 19), inconsistent zero ED intake (0.0 L despite reported ED consumption in general; *n* = 805), or implausibly high ED intake (more than 5 L on a consumption day; *n* = 52).

**Table 3:** Caffeine intake from other caffeinated beverages and from all caffeinated beverages across ED consumption groups

	Caffeine intake from other caffeinated beverages ^a^ (excluding EDs)			Caffeine intake from all caffeinated beverages (including EDs)		
	Don‘t know/ no answer	Usual daily caffeine intake [mg/day]	Usual daily weight-adjusted caffeine intake [mg/kg BW/day]	Inconsistent answers ^b^	Usual daily caffeine intake [mg/day]	Usual daily weight-adjusted caffeine intake [mg/kg BW/day]
Non-consumers of EDs (*n* = 1685)	61	18.1 (5.0–58.7)	0.3 (0.1–1.0)	–	18.1 (5.0–58.7)	0.3 (0.1–1.0)
Occasional ED consumers (*n* = 1714)	24	34.8 (11.4–84.6)	0.5 (0.2–1.3)	771	46.6 (21.4–97.9)	2.5 (1.7–4.4)
Moderate ED consumers (*n* = 875)	11	56.8 (20.6–118)	0.8 (0.3–1.8)	91	114 (62.2–206)	3.4 (2.1–5.4)
Regular ED consumers (*n* = 80)	1	82.1 (24.9–220)	1.2 (0.3–2.8)	10	269 (38.0–498)	4.9 (2.7–9.2)
Chronic low ED consumers (*n* = 252)	2	82.6 (33.9–168)	1.2 (0.5–2.5)	8	187 (127–282)	3.1 (2.3–4.5)
Chronic high ED consumers (*n* = 287)	4	126 (52.2–237)	1.9 (0.8–3.5)	23	428 (306–631)	7.3 (5.7–11.1)

Variables expressed as median (IQR)

^a^ Other caffeinated beverages include coffee drinks, black and green tea, drinking chocolate, cola and mate soft drinks, iced tea, and pre-workout boosters

^b^ Inconsistent answers included “don’t know/no answer” (either in ED or caffeinated beverages questions; *n* = 46), inconsistent zero ED intake (0.0 L despite reported ED consumption in general; *n* = 805), or implausibly high ED intake (more than 5 L on a consumption day; *n* = 52)

### Sociodemographic and lifestyle characteristics across ED consumption groups

The distribution of ED consumption groups did not differ considerably between boys and girls (Supplementary Figure S3, Supplementary Tables S4 and S5). However, adolescents identifying as diverse showed a different distribution of ED consumption groups, with a higher proportion of chronic ED consumers compared with both boys and girls. Across age categories, the distribution of ED consumption groups was similar, with chronic high ED consumers consistently ranging between 4.6 and 6.7% (Supplementary Figure S4, Supplementary Table S6). Differences were only observed in the proportion of non-consumers, which was highest among 15-year-olds (41.9%). Non-consumption was also more common in grammar schools (44.4%, 95% CI: 41.9–47.0) than in secondary (31.9%, 95% CI: 30.0–33.8) or vocational schools (26.8%, 95% CI: 24.3–29.4), whereas chronic high ED consumption was most prevalent at vocational schools (9.1%, 95% CI: 7.4–10.8) compared with grammar schools (2.7%, 95% CI: 1.9–3.5) (Supplementary Figure S5, Supplementary Table S7).

Detailed analyses regarding BMI percentile categories noticed that 73.9% (95% CI: 72.6–75.1) of the entire study population were classified as normal weight (P10–P90), 8.2% (95% CI: 7.4–8.9) as underweight (< P10), 9.9% (95% CI: 9.0–10.7) as overweight (> P90–P97), and 8.1% (95% CI: 7.3–8.9) as obese (> P97) (Supplementary Figure S6, Supplementary Table S8). Non-consumers showed the lowest median (IQR) BMI of 20.8 kg/m² (19.0–23.2) as well as the lowest proportions within the overweight and obesity BMI percentile categories compared with all other ED consumer groups (Table 1, Supplementary Figure S6, Supplementary Table S8). The highest prevalence of obesity was observed among moderate (12.1%, 95% CI: 10.0–14.3) and chronic low ED consumers (11.5%, 95% CI: 7.6–15.4), whereas the lowest was observed among non-consumers (5.3%, 95% CI: 4.3–6.4). Underweight was most common among non-consumers (10.9%, 95% CI: 9.4–12.4), but was also observed among chronic high ED consumers (7.7%, 95% CI: 4.6–10.7).

Physical activity also varied across consumption groups, with the most pronounced differences between non-consumers and chronic ED consumers. Never exercising was more prevalent among chronic low (13.1%) and chronic high ED consumers (13.2%) compared with non-consumers (8.6%) (Table 1). Conversely, exercising two to six times per week was slightly more frequent among non-consumers (50.3%) than among chronic low (46.4%) and chronic high ED consumers (42.2%).

Potentially harmful lifestyle behaviors were more prevalent with higher ED consumption, with the proportion of smokers (including use of cigarettes, shisha, e-cigarettes, e-shisha, or tobacco heaters) increased across ED consumption groups (Table 1, Supplementary Figure S7, Supplementary Table S9). The choice of smoking products also differed: the use of shisha, e-cigarettes, e-shisha, or tobacco heaters was more prevalent among ED consumers than among non-consumers, with usage increasing across ED consumption groups. In contrast, the overall prevalence of conventional cigarette use appeared comparable across ED consumption groups. However, the frequency differed, with the proportion of daily cigarette smoking increasing from 40.6% among non-consumers to 74.7% among chronic high ED consumers, who also reported smoking a higher number of cigarettes per day (Supplementary Table S10). A similar trend was observed for cannabis smoking (herb and resin): 10.3% of non-consumers reported ever smoking cannabis, compared with almost 50% among chronic high ED consumers, of whom 7.0% reported smoking daily cannabis smoking (Table 1).

Alcohol consumption was also more common among ED consumers, with more than 70% reporting lifetime alcohol consumption compared with 54.5% of non-consumers (Table 1). ED consumers, and especially chronic high ED consumers, reported both drinking alcohol more frequently in the past 12 months and consuming larger amounts on single occasions of four or more glasses (Table 1, Supplementary Table S11).

**Fig. 3:**
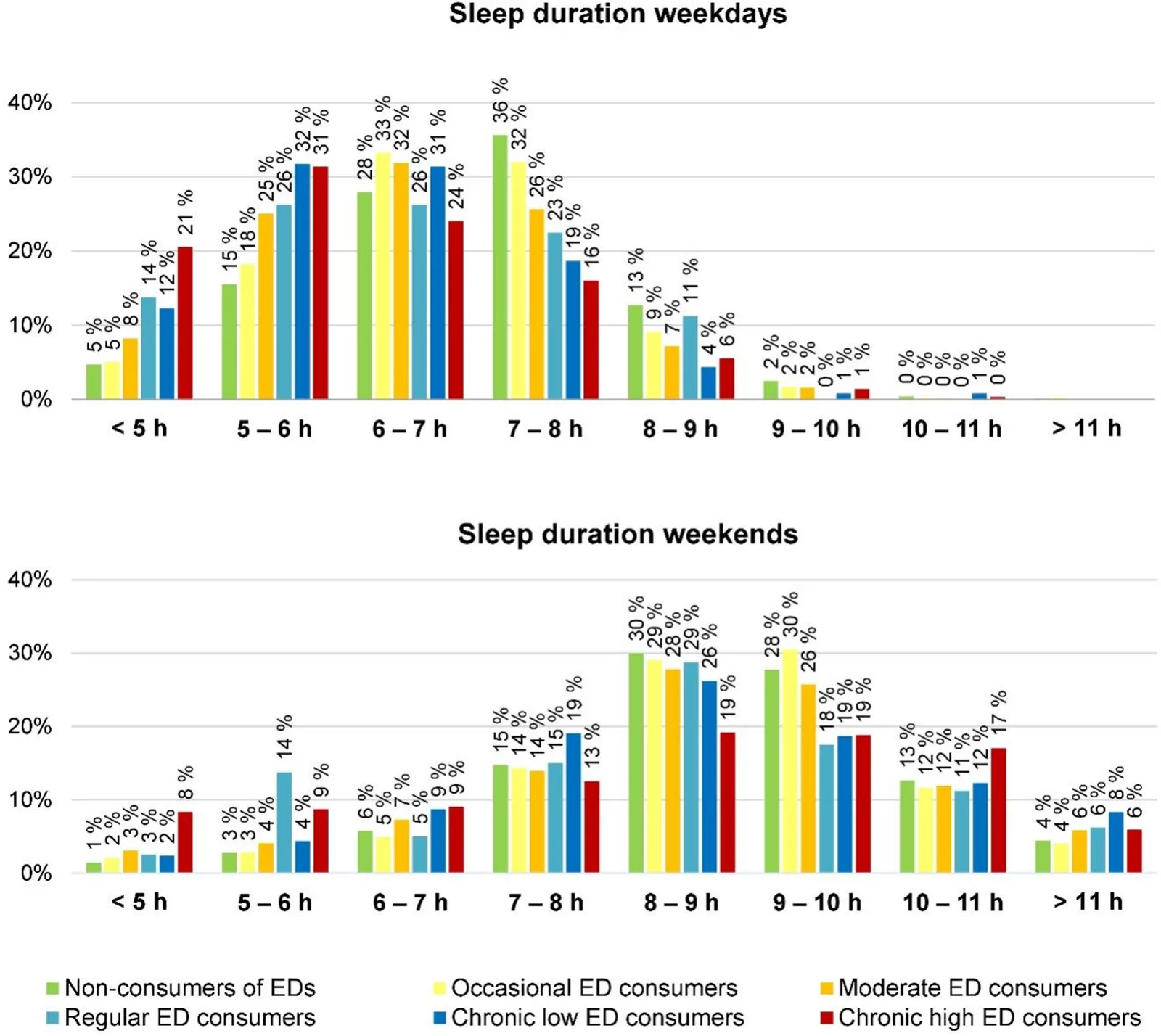
Distribution of ED consumption groups across sleep duration categories on weekdays and weekends

ED consumption was also linked to sleep behavior, with the proportion of adolescents reporting short sleep durations (< 5 h and 5–6 h) on weekdays increased across ED consumption groups, while sleep of 7–8 h became less frequent (Fig. 3). As expected, sleep duration was longer on weekends for most participants, but very short sleep (< 5 h) was most prevalent among chronic high ED consumers. Similarly, sleep disturbances were likewise more prevalent with increasing ED consumption, reported by 28.7% of non-consumers and 58.2% of chronic high ED consumers (Table 1). Among those with sleep disturbances, difficulties falling asleep were consistently reported by around three-quarters of the participants in each consumption group. Problems sleeping through the night were more prevalent among chronic low (59.8%) and chronic high ED consumers (55.7%) than among non-consumers (40.1%).

### Leisure activities and media consumption across ED consumption groups

**Fig. 4:**
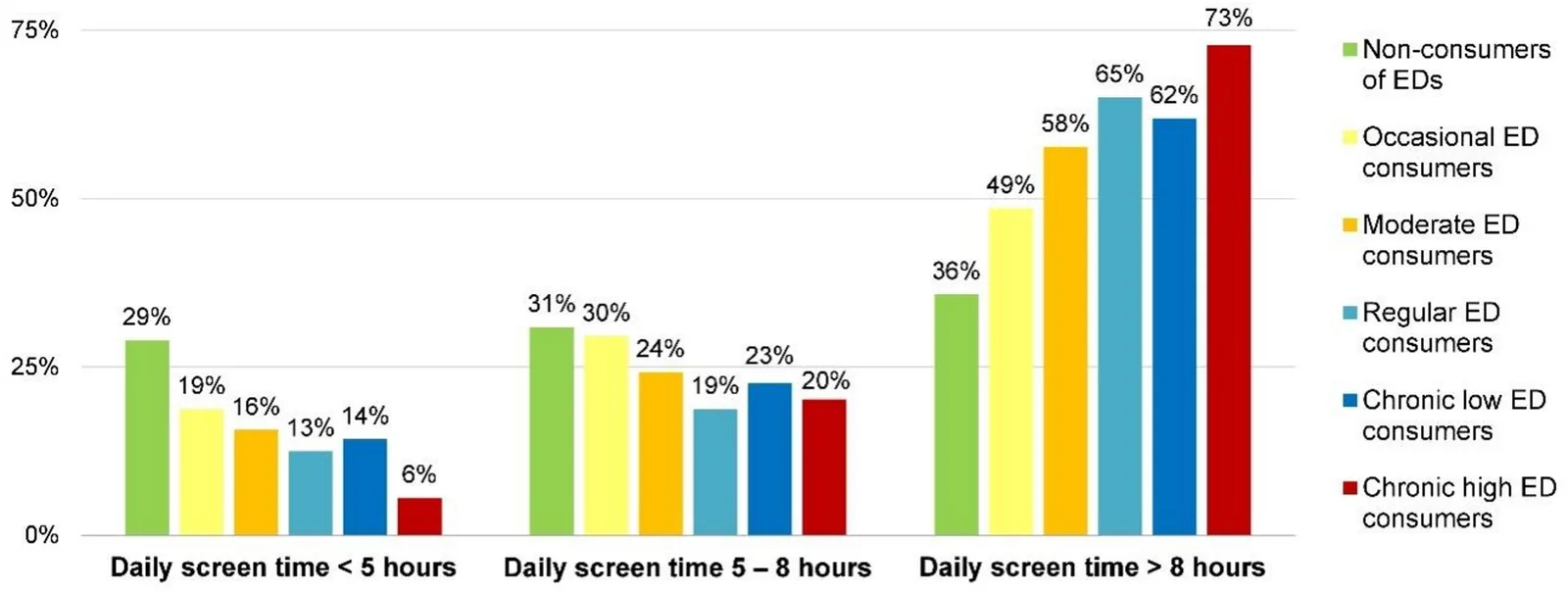
Distribution of ED consumption groups across daily screen time categories

Differences in leisure behavior and media consumption were observed between non-consumers and ED consumers, particularly in the domains of social interaction and digital media use (Supplementary Table S12 and S13). Social interaction increased with higher ED consumption, both through daily personal meetings with friends or other people outside of school (24.7% of chronic high ED consumers vs. 4.7​% of non-consumers, Supplementary Table S12) and through the use of messenger services for more than three hours per day (35.9% of chronic high ED consumers vs. 8.7% of non-consumers, Supplementary Table S13). Similar trends were observed for video-based communication with friends and other people. These behavioral differences across the ED consumption groups were also reflected in the reported durations of digital media use, including gaming for more than three hours per day (43.6% of chronic high ED consumers vs. 20.8% of non-consumers) and social network use for more than three hours per day (36.6% of chronic high ED consumers vs. 11.0% of non-consumers) (Supplementary Table S13). An increase in media use was also evident across ED consumption groups in overall daily screen time, with 72.8% (95% CI: 67.7–77.9) of chronic high ED consumers reporting more than eight hours of screen time per day, compared with 35.8% (95% CI: 33.5–38.1) of non-consumers (Fig. 4, Supplementary Table S14). By contrast, adolescents in all ED consumption groups were less likely than non-consumers to spend time reading or taking part in musical activities such as playing an instrument, singing, or composing music (Supplementary Tables S12 and S13).

## Discussion

The EDKAR-study provides a comprehensive descriptive overview of ED consumption and associated lifestyle factors among German adolescents aged 15 to 18 years. With a study population of 4893 participants, this study represents one of the largest and most detailed investigations of ED consumption in this vulnerable group to date. Our findings highlight the high prevalence of ED consumption among adolescents and its association with a variety of harmful lifestyle behaviors, including daily caffeine intake from all sources, smoking, alcohol consumption (also in combination with EDs), short sleep durations, and excessive daily screen time.

The consumption of EDs is a widespread and growing public health concern among adolescents, with global prevalence estimates showing substantial regional variation [3, 4]. A recent systematic review and meta-analysis conducted by Aonso-Diego et al. reported a worldwide pooled lifetime prevalence of 57.7% and a past-year prevalence of 44.5% of ED consumption among adolescents aged 12 to 18 years [4]. Published data on German adolescents showed a lifetime prevalence of ED consumption of 61.7% in a study including participants aged 7 to 17 years [15] and a prevalence of 73% in a study including European adolescents aged 15 to 18 years [3]. A more recently published survey from Germany (2024) found that 57% of adolescents aged 14 to 18 years had consumed EDs [6]. Similarly, the present EDKAR-study indicates that 71.9% of adolescents reported having consumed EDs at least once in their lifetime, and 65.6% reported ED consumption within the past 12 months. Furthermore, 12.6% of participants reported consuming EDs at least four times per week (Supplementary Figure S1), which is higher than the 8% reported by Zucconi et al. among European adolescents aged 10 to 18 years [3]. By contrast, a recent German survey found lower consumption frequencies, with 19% of participants reporting ED consumption several times a month, and 8% several times a week [6]. Daily ED consumption was also less common in the German survey than in the EDKAR-study (2% vs. 3.9%) [6]. Regarding chronic ED consumption among all ED consumers, Zucconi et al. identified 11% of European adolescents aged 15–18 years as “high chronic consumers” of EDs, defined as consuming EDs at least four times per week over the past 12 months, with an average intake of seven liters per month (approximately one ED can per day) [3]. Similar to the EFSA definition [3], regular, chronic low, and chronic high ED consumers together represented a notable 19.3% of all ED consumers in the EDKAR-study. Among them, 8.9% were classified as chronic high ED consumers, defined by ED consumption at least four times a week within the past 12 months while exceeding the daily caffeine threshold of 3 mg per kg BW through ED consumption alone.

Regarding caffeine intake from EDs, chronic high ED consumers reached a median intake of 262 mg/day, which was substantially higher than the median intake observed among all ED consumers (26.5 mg/day). In the study by Zucconi et al., caffeine intake from EDs ranged from 23.5 mg/day among total ED consumers to 75.1 mg/day among chronic high ED consumers [3]. The substantially higher caffeine intake from EDs observed in the EDKAR-study compared with Zucconi et al. in 2013 [3] might indicate that caffeine exposure from EDs has increased over the past decade. Referring to the same study, total caffeine exposure from all dietary sources ranged from 184.9 mg/day among total ED consumers to 477 mg/day among chronic high ED consumers [3]. In the EDKAR-study, the corresponding caffeine intake from all caffeinated beverages (including EDs) was lower among all ED consumers (104.8 mg/day), but remained high among chronic high ED consumers (428 mg/day). Interestingly, the EDKAR-study showed an increase in caffeine intake from other caffeine-containing beverages across the ED consumption groups. This finding is noteworthy, as it indicates that adolescents with a high caffeine intake from EDs also tend to consume considerable amounts of caffeine from other caffeinated beverages.

Importantly, the present study revealed that not only chronic high ED consumption but also acute high intake of EDs on a single consumption day was frequently observed even among adolescents with lower consumption frequencies. Overall, 11.6% of all ED consumers reported drinking more than one liter of EDs on a consumption day. Zucconi et al. reported a prevalence of 17% for high acute ED consumption among German adolescents aged 10–18 years, defined as consuming at least 1.065 L of EDs per single consumption session [3]. These findings suggest that there is a substantial potential for binge-like ED consumption, even among adolescents who do not frequently consume EDs. Binge-like ED consumption leads to excessively high caffeine exposure, which is particularly relevant because caffeine is the main bioactive compound responsible for the stimulating effects of EDs [7, 16]. Such consumption behaviors are concerning, especially given the evidence linking acute high ED consumption with severe cardiovascular adverse effects, including elevated heart rate, arrhythmias, increased blood pressure, and QTc prolongation [2, 8, 12, 17–22]. Although the majority of the literature suggests that typical, moderate caffeine consumption in children and adolescents is relatively safe, acute caffeine intake levels exceeding 400 mg (equivalent to approximately 1.25 L of EDs per day) cause physiological (e.g., tachycardia, hypertension, tremor), psychological (e.g., anxiety, sleep disturbances), or behavioral harm (e.g., increased substance use) [23]. Beyond caffeine itself, there is an ongoing discussion that other ED ingredients such as taurine, guarana, and glucuronolactone may enhance or modify the effects of caffeine [1, 2]. Particularly concerning is the fact that 28.7% of all ED consumers in the EDKAR-study reported at least one acute adverse effect after ED consumption within the past 12 months. Moreover, adverse effects were reported more frequently among adolescents with higher ED consumption, with 50.2% of chronic high ED consumers having experienced adverse effects. As comprehensively summarized in a recent systematic review on ED-related adverse health events in children and adolescents, the most frequently reported acute symptoms in minors include tachycardia, palpitations, chest pain, insomnia, anxiety, stress, and headaches [12]. Surprisingly, a high percentage of individuals who experienced adverse effects continued to consume EDs. This may indicate a low perception of risk and a possible normalization of these symptoms among adolescents. Similar findings pointing to low risk awareness and continued ED consumption despite adverse effects have been reported among university students in Italy [24]. Another possible explanation for the continued consumption of EDs despite adverse effects is a lack of knowledge about their ingredients and physiological effects. In fact, Alabbad et al. showed that many young consumers are unaware of the specific ingredients in EDs and their potential health consequences [25]. In a study by Flotta et al., only 13% of adolescents recognized that drinking EDs leads to an equivalent caffeine intake as drinking a cup of coffee, while a substantial proportion believed that EDs are comparable to soft drinks or sports beverages [26]. This fundamental misunderstanding may contribute to the normalization of ED consumption and the underestimation of associated health risks resulting from high caffeine intake, indicating that general warnings (e.g., “high caffeine content”) on product labels are insufficient to raise adequate risk awareness among adolescents. Young consumers should be informed about the potential adverse effects associated with high ED consumption so that they can make informed decisions.

As awareness and knowledge of the ED-related health risks may influence consumption behavior, the sociodemographic differences in ED consumption observed in the EDKAR-study are particularly noteworthy. The EDKAR-study found no differences in ED consumption between girls and boys across ED consumer groups. This finding differs from results reported among adolescents worldwide, where boys were found to consume significantly more EDs than girls [9, 11]. However, when considering gender diversity, the EDKAR-study revealed that adolescents who identify as gender diverse are a potentially vulnerable group, showing a higher proportion of high ED consumption than boys or girls. Age also appeared to be a relevant factor, with the highest proportion of non-consumers was found among 15-year-olds, and ED consumption tended to increase with age. A similar trend has been reported in international studies, which have found higher ED consumption among older adolescents than younger ones [4, 9]. Differences in ED consumption were also observed according to BMI status, with a higher proportion of adolescents in the ED consumer groups being classified as overweight or obese compared with non-consumers. This finding may be linked to the behavioral characteristics of chronic ED consumers, who reported lower levels of physical activity and a stronger preference for EDs containing sugar. Similar associations have been reported in young adults, where sugar-sweetened beverage intake and low physical activity were identified as correlates of being overweight [27]. However, it should be noted that underweight adolescents were also represented among the frequent ED consumers. The EDKAR-study also observed differences in ED consumption behaviors across educational environments, suggesting that the immediate social context of adolescents, particularly the school environment, may play an important role in ED consumption behaviors. The highest proportion of non-consumers was found among pupils attending grammar schools, whereas the proportion of chronic low and chronic high ED consumers was considerably higher among those at vocational schools. Differences in school environments may reflect variations in group norms, social behaviors, and health awareness that are associated with ED consumption behavior. This interpretation is supported by a previous study showing that better knowledge of ED ingredients and their potential health risks is associated with lower consumption rates [10].

The relevance of health awareness may not be limited to ED consumption alone. Systematic reviews and recent studies have identified a common pattern of ED consumption and polysubstance use among adolescents, including alcohol, tobacco, and soft and hard drugs [1, 5, 9, 16, 28–30]. Moreover, a longitudinal study demonstrated that early ED consumption is associated with an increased likelihood of later substance use, including alcohol, tobacco, cannabis, and other products such as e-cigarettes or hookah [15]. This proposed co-occurrence of substance use behaviors was also observed among adolescents in the EDKAR-study. In particular, alcohol consumption was substantially higher across all ED consumer groups compared with non-consumers. Furthermore, in the present study, the combination of EDs with alcohol was also more prevalent among adolescents with higher ED consumption. Among all ED consumers, 41% reported mixing EDs with alcohol, a proportion that increased to 61% among chronic high ED consumers. These rates are lower than earlier findings, which reported a co-consumption rate of 61% among German adolescents consuming EDs [3]. The behavior of mixing EDs with alcohol is particularly concerning given the well-documented risk that caffeine can mask the adverse effects of alcohol, which may increase the likelihood of excessive alcohol intake, injury, and substance misuse [2, 10]. Furthermore, the combination of EDs and alcohol has been associated with dehydration, increased heart rate, arrhythmias, elevated blood pressure, and impaired perception of intoxication [10]. Additionally, rates of smoking (including cigarettes, e-cigarettes, shisha, e-shisha, and tobacco heaters) as well as smoking cannabis (herb and resin) increased consistently across ED consumer groups. Among chronic high ED consumers, more than half reported current smoking, and nearly half had smoked cannabis. These findings are in line with previous research, which showed strong associations between ED consumption and risky substance use behaviors [1, 5, 9, 10, 28]. Another important health risk associated with ED consumption concerns sleep behavior, as clearly demonstrated in the EDKAR-study. Adolescents with higher ED consumption reported shorter sleep durations and more frequent difficulties staying asleep. These findings are consistent with recent studies showing that shorter sleep duration is associated with a higher likelihood of ED consumption among adolescents [31, 32]. Notably, the EDKAR-study showed that the expected compensatory effect of longer weekend sleep was not consistently observed among chronic high ED consumers. A substantial number of adolescents in this subgroup continued to report sleep times under 6 h per night even on weekends. This finding may indicate that frequent ED consumption is associated with insufficient natural sleep recovery, even on weekends, which is consistent with the well-documented sleep-disturbing effects of caffeine [7, 23]. However, whether and to what extent the consumption of EDs triggers these harmful lifestyle behaviors (e.g., alcohol, smoking, and sleep) or vice versa, or to what extent these factors may be influenced by a more risk-taking attitude, cannot be determined and is beyond the scope of this study.

Additional lifestyle factors examined in the EDKAR-study, such as screen time and social interaction, showed a noticeable increase across ED consumer groups. Adolescents with higher ED consumption reported spending more time with friends and using screen-based media. This trend was observed not only in the frequency of in-person social interactions but also in the use of digital communication channels such as messaging apps and video calls. These findings are in line with the results of a study of young adults, in which 29.1% of participants reported consuming EDs to socialize [24]. Other studies suggest that social and digital environments may promote both the initiation and the ongoing consumption of EDs, particularly through peer influence, recommendations from friends and family, and targeted marketing strategies [9, 33]. Such strategies, including the way EDs are marketed and presented, rarely convey that these beverages may pose adverse health effects to young consumers. Accordingly, some European countries have introduced age restrictions on ED sales, whereas no such regulations currently exist in Germany, making these products easily accessible to children and adolescents [34, 35].

The strengths of our EDKAR-study include its comprehensive characterization of different ED consumer groups. Notable methodological advantages include the large sample size, the systematic classification of ED consumer groups, and the wide-ranging assessment of ED consumption behaviors, as well as associated lifestyle factors among German adolescents. While the EDKAR-study offers valuable insights, several limitations must be acknowledged. The cross-sectional design of the EDKAR-study, along with the descriptive analysis of the present results, does not allow any causal conclusions regarding whether ED consumption contributes to the observed lifestyle characteristics or whether adolescents with these characteristics are more likely to consume EDs. Furthermore, ED consumption may also occur as a concomitant or compensatory behavior in relation to some of these characteristics. In addition, the generalizability of the findings is limited, as data were collected solely in urban Berlin and were restricted to adolescents aged 15 to 18 years. Another limitation concerns the use of a self-administered online questionnaire, which may introduce recall and social desirability bias, potentially leading to under- or overreporting of consumption frequencies and other sensitive behaviors in this vulnerable group. This is particularly relevant when quantifying how frequently adolescents consume substances such as EDs, alcohol, cigarettes, or cannabis, as participants may report inaccurate consumption frequencies due to memory bias, social pressure, or shame. These biases may also have contributed to the considerable proportion of inconsistent ED intake responses, particularly among occasional ED consumers. Some participants may have reported zero ED intake because they consumed only small amounts of EDs, whereas others may have provided incomplete or inaccurate information. However, the underlying reasons for these inconsistent responses could not be determined from the available data.

## Conclusion

The EDKAR-study highlights the high prevalence of ED consumption among adolescents in Germany and its association with various lifestyle characteristics, including increased caffeine intake, higher rates of smoking and alcohol consumption, short sleep durations, extended daily screen time and media use, as well as a high proportion of reported adverse effects following ED consumption. The frequent co-occurrence of ED consumption and harmful lifestyle behaviors, particularly among chronic high ED consumers, highlights the need for early and targeted prevention strategies that address ED consumption, as well as alcohol consumption, smoking, and sleep hygiene. Educational efforts should raise adolescents’ awareness of the potential adverse health effects of ED consumption and of the harmful lifestyle behaviors that co-occur with high ED consumption, encouraging them to critically reflect on their consumption behaviors while promoting healthier alternatives.

## Supplementary Information

Below is the link to the electronic supplementary material.

Supplementary Material 1

## Data Availability

The data used in the present study will not be made publicly available, but they are accessible by contacting the corresponding author.
