# Supplementary material for: Energy drink consumption and associated lifestyle factors among German adolescents: findings from the EDKAR-study
Source: Eur J Nutr. 2026 Nov 11;65(8):300. doi: 10.1007/s00394-026-04153-x (PMC13755080; doi:10.1007/s00394-026-04153-x)
Supplement: Supplementary file 1 [file 394_2026_4153_MOESM1_ESM.docx]

**Supplementary Material to:**

**Energy drink consumption and associated lifestyle factors among German adolescents:
findings from the EDKAR-study**

European Journal of Nutrition

Maria J. Pie^1,2^, Sven Knüppel^1^, Britta Nagl^1^, Anke Ehlers^1^, Fabian Spinka^3,4,5^, Andrea Deichl^3,4,5^, Frank Edelmann^3,4,5^, Cornelia Weikert^1,2^, *Juliane Menzel^1,2^

^1^ Department Food and Feed Safety in the Food Chain, German Federal Institute for Risk Assessment, Berlin, Germany

^2^ Institute of Social Medicine, Epidemiology and Health Economics, Charité – University Medical Center Berlin, Corporate Member of Freie Universität Berlin and Humboldt-Universität zu Berlin, Berlin, Germany

^3^ German Center for Cardiovascular Research (DZHK), Partner Site Berlin, Berlin, Germany

^4^ Department of Cardiology, Angiology and Intensive Care Medicine, Deutsches Herzzentrum der Charité (DHZC) – Medical Heart Center of Charité and German Heart Institute Berlin, Berlin, Germany

^5^ Charité – University Medical Center Berlin, Corporate Member of Freie Universität Berlin and Humboldt-Universität zu Berlin, Berlin, Germany.

***Corresponding author:**

PD Dr Juliane Menzel

German Federal Institute for Risk Assessment

Max-Dohrn-Straße 8-10

10589 Berlin, Germany

Juliane.Menzel@bfr.bund.de

**Supplementary Figures**

**Supplementary Fig. S1** Reported frequency of ED consumption during the past 12 months among all participants (n=4893)

**Supplementary Fig. S2** Reported duration of ED consumption among all ED consumers (n=3208)

**Supplementary Fig. S3** Distribution of ED consumption groups within gender categories

**Supplementary Fig. S4** Distribution of ED consumption groups within age categories

**Supplementary Fig. S5** Distribution of ED consumption groups within school type categories

**Supplementary Fig. S6** Distribution of ED consumption groups across BMI percentile categories

**Supplementary Fig. S7** Distribution of ED consumption groups across smoking status categories. Smoking includes the use of cigarettes, shisha, e-cigarettes, e-shisha, or tobacco heaters

**Supplementary Tables**

Supplementary Table S1 Comparison of baseline characteristics among the three non-consumer subgroups.

|  | **All**  **non-consumers (n=1685)** | **Lifelong**  **non-consumers**  **(n=643)** | **Tried-once**  **non-consumers (n=734)** | **Past-year non-consumers (n=308)** |
| --- | --- | --- | --- | --- |
| **Gender** |  |  |  |  |
| Male | 52.2% (880) | 51.0% (328) | 50.4% (370) | 59.1% (182) |
| Female | 46.9% (790) | 48.7% (313) | 48.5% (356) | 39.3% (121) |
| Diverse | 0.9% (15) | 0.3% (2) | 1.1% (8) | 1.6% (5) |
| **Age** [years] | 16.0 ± 1.0 | 15.9 ± 0.9 | 16.1 ± 1.0 | 16.3 ± 1.0 |
| **BMI** [kg/m^2^] | 20.8 (19.0–23.2) | 20.7 (18.8–23.1) | 20.7 (18.9–23.2) | 21.4 (19.6–23.5) |
| **School type** |  |  |  |  |
| Grammar school | 38.2% (644) | 40.0% (257) | 39.2% (288) | 32.1% (99) |
| Secondary school | 43.8% (738) | 46.0% (296) | 40.3% (296) | 47.4% (146) |
| Vocational school center | 18.0% (303) | 14.0% (90) | 20.4% (150) | 20.5% (63) |
| **Country of birth** |  |  |  |  |
| Germany | 86.3% (1454) | 88.3% (568) | 86.9% (638) | 80.5% (248) |
| Other country | 13.5% (227) | 11.5% (74) | 12.9% (95) | 18.8% (58) |
| Don’t know/no answer | 0.2% (4) | 0.2% (1) | 0.1% (1) | 0.6% (2) |
| **Physical activity** |  |  |  |  |
| Never | 8.6% (145) | 9.5% (61) | 8.7% (64) | 6.5% (20) |
| Less than once a week | 9.1% (154) | 9.8% (63) | 9.4% (69) | 7.1% (22) |
| Once a week | 17.6% (296) | 18.2% (117) | 17.2% (126) | 17.2% (53) |
| 2-6 times a week | 50.3% (848) | 48.2% (310) | 50.5% (371) | 54.2% (167) |
| Daily | 12.0% (203) | 12.0% (77) | 11.9% (87) | 12.7% (39) |
| Don’t know/no answer | 2.3% (39) | 2.3% (15) | 2.3% (17) | 2.3% (7) |
| **Smoking status** ^a^ |  |  |  |  |
| Smoker | 5.6% (94) | 1.6% (10) | 6.7% (49) | 11.4% (35) |
| Ex-smoker | 6.8% (115) | 2.5% (16) | 7.9% (58) | 13.3% (41) |
| Non-smoker | 85.8% (1445) | 94.6% (608) | 83.1% (610) | 73.7% (227) |
| Don’t know/no answer | 1.8% (31) | 1.4% (9) | 2.3% (17) | 1.6% (5) |
| **Smoking cannabis (herb and resin)** |  |  |  |  |
| Yes | 10.3% (174) | 2.3% (15) | 13.2% (97) | 20.1% (62) |
| No | 88.1% (1485) | 96.9% (623) | 85.0% (624) | 77.3% (238) |
| Don’t know/no answer | 1.5% (26) | 0.8% (5) | 1.8% (13) | 2.6% (8) |
| If yes, daily cannabis smokers | 0.2% (3) | – | 0.4% (3) | – |
| **Alcohol consumption** |  |  |  |  |
| Yes | 54.5% (918) | 39.5% (254) | 63.6% (467) | 64.0% (197) |
| No | 44.2% (744) | 59.4% (382) | 34.7% (255) | 34.7% (107) |
| Don’t know/no answer | 1.4% (23) | 1.1% (7) | 1.6% (12) | 1.3% (4) |
| If yes, Glasses of alcohol per week ^b^ | 0.2 (0.1–1.7) | 0.1 (0.1–0.6) | 0.3 (0.1–1.7) | 0.3 (0.1–2.0) |
| If yes, ≥ 4 glasses on one occasion | 7.8% (132) | 3.0% (19) | 10.6% (78) | 11.4% (35) |
| **Sleep disturbances** |  |  |  |  |
| Yes | 28.7% (484) | 26.3% (169) | 30.1% (221) | 30.5% (94) |
| No | 65.2% (1098) | 66.6% (428) | 64.7% (475) | 63.3% (195) |
| Don’t know/no answer | 6.1% (103) | 7.2% (46) | 5.2% (38) | 6.2% (19) |
| If yes, difficulty falling asleep ^c^ | 77.7% (376) | 78.1% (132) | 78.7% (174) | 74.5% (70) |
| If yes, difficulty staying asleep ^c^ | 40.1% (194) | 39.6% (67) | 37.1% (82) | 47.9% (45) |

Variables expressed as percentage (n), mean (± SD), or median (IQR).

^a^ Smoking included consumption of cigarettes, shisha, e-cigarettes, e-shisha, or tobacco heaters.

^b^ Missings: n = 178.

^c^ Multiple answers possible.

Supplementary Table S2 Usual daily ED and caffeine intake stratified by intake quintiles

| **Usual daily intake of EDs  [ml/day]** | **Q1 (n=340)**  4.0 (4.0–8.0) | **Q2 (n=596)**  22.6 (20.5–35.8) | **Q3 (n=482)**  88.6 (71.5–107) | **Q4 (n=431)**  215 (188–285) | **Q5 (n=483)**  712 (500–1045) |
| --- | --- | --- | --- | --- | --- |
| **Usual daily caffeine intake from EDs  [mg/day]** | **Q1 (n=340)**  1.3 (1.3–2.6) | **Q2 (n=596)**  7.2 (6.6–11.4) | **Q3 (n=482)**  28.3 (22.9–34.3) | **Q4 (n=431)**  68.6 (60.3–91.4) | **Q5 (n=483)**  228 (160–335) |
| **Usual daily weight-adjusted caffeine intake from EDs  [mg/kg BW/day]** | **Q1 (n=466)**  1.1 (0.9–1.1) | **Q2 (n=476)**  1.4 (1.3–1.5) | **Q3 (n=477)**  2.9 (1.8–2.3) | **Q4 (n=467)**  3.2 (2.9–3.7) | **Q5 (n=466)**  7.0 (5.3–10.5) |

Data are presented as median (IQR). Quintiles were calculated separately for each intake variable among ED consumers, with Q1 representing the lowest and Q5 the highest quintile of intake. Quintile sizes may differ due to identical intake values. Analyses included 2332 ED consumers after excluding inconsistent answers comprising self-reported ED consumption of 0 ml per day (n = 805), more than 5 liter per day (n = 52), or “don’t know/no answer” (n = 19).

**Supplementary Table S3** Reasons for ED consumption stratified by ED consumption groups (multiple responses possible)

|  | **Overall  ED consumers  (n=3208)** | **Occasional  ED consumers  (n=1714)** | **Moderate  ED consumers  (n=875)** | **Regular  ED consumers  (n=80)** | **Chronic low  ED consumers  (n=252)** | **Chronic high  ED consumers  (n=287)** |
| --- | --- | --- | --- | --- | --- | --- |
| Taste | 68.0% (2181) | 19.1% (322) | 60.7% (1041) | 75.0% (656) | 66.3% (53) | 80.2% (202) |
| To wake up | 45.6% (1462) | 20.2% (340) | 39.4% (675) | 48.5% (424) | 48.8% (39) | 56.8% (143) |
| Stay awake longer | 35.3% (1131) | 18.8% (316) | 32.4% (556) | 35.2% (308) | 37.5% (30) | 36.9% (93) |
| Enhance performance | 24.2% (775) | 9.4% (158) | 19.5% (334) | 27.3% (239) | 31.3% (25) | 30.2% (76) |
| Improve concentration | 20.6% (661) | 6.5% (109) | 15.1% (258) | 24.2% (212) | 18.8% (15) | 28.2% (71) |
| Friends drinking ED | 11.6% (373) | 8.1% (136) | 10.7% (184) | 11.5% (101) | 5.0% (4) | 9.1% (23) |
| Variety of Flavors | 11.6% (372) | 2.7% (46) | 9.2% (158) | 12.2% (107) | 13.8% (11) | 11.9% (30) |
| Positive effects | 8.1% (261) | 2.0% (34) | 4.7% (81) | 9.1% (80) | 17.5% (14) | 12.7% (32) |
| Parents / siblings | 2.7% (85) | 1.2% (21) | 2.5% (43) | 2.3% (20) | 1.3% (1) | 2.4% (6) |
| ED advertising | 0.8% (27) | 1.0% (17) | 0.5% (9) | 0.7% (6) | 1.3% (1) | 1.2% (3) |
| Don´t know/No answer | 3.2% (104) | 4.4% (75) | 1.8% (16) | 3.8% (3) | 1.6% (4) | 2.1% (6) |

**Supplementary Table S4** 95% confidence intervals for the distribution of ED consumption groups within gender categories

|  | **Male  (n=2633)** | **Female  (n=2193)** | **Diverse  (n=67)** |
| --- | --- | --- | --- |
| Non-consumers of EDs | 33.4% (31.6–35.2) | 36.0% (34.0–38.0) | 22.4% (12.4–32.4) |
| Occasional ED consumers | 34.8% (32.9–36.6) | 35.4% (33.4–37.4) | 34.3% (23.0–45.7) |
| Moderate ED consumers | 19.1% (17.6–20.6) | 16.4% (14.9–18.0) | 19.4% (9.9–28.9) |
| Regular ED consumers | 1.9% (1.3–2.4) | 1.4% (0.9–1.9) | – |
| Chronic low ED consumers | 5.2% (4.4–6.1) | 4.9% (4.0–5.8) | 10.4% (3.1–17.8) |
| Chronic high ED consumers | 5.7% (4.8–6.6) | 5.8% (4.9–6.8) | 13.4% (5.3–21.6) |

**Supplementary Table S5** 95% confidence intervals for the distribution of ED consumption groups across gender categories

|  | **Overall  population (n=4893)** | **Non-consumers  of EDs  (n=1685)** | **Occasional  ED consumers  (n=1714)** | **Moderate  ED consumers  (n=875)** | **Regular  ED consumers  (n=80)** | **Chronic low  ED consumers  (n=252)** | **Chronic high  ED consumers  (n=287)** |
| --- | --- | --- | --- | --- | --- | --- | --- |
| Male | 53.8% (52.4–55.2) | 52.2% (49.8–54.6) | 53.4% (51.0–55.7) | 57.4% (54.1–60.6) | 61.3% (50.6–71.9) | 54.4% (48.2–60.5) | 52.3% (46.5–58.0) |
| Female | 44.8% (43.4–46.2) | 46.9% (44.5–49.3) | 45.3% (42.9–47.6) | 41.1% (37.9–44.4) | 38.8% (28.1–49.4) | 42.9% (36.7–49.0) | 44.6% (38.8–50.4) |
| Diverse | 1.4% (1.0–1.7) | 0.9% (0.4–1.3) | 1.3% (0.8–1.9) | 1.5% (0.7–2.3) | – | 2.8% (0.7–4.8) | 3.1% (1.1–5.2) |

**Supplementary Table S6** 95% confidence intervals for the distribution of ED consumption groups within age categories

|  | **15 years**  **(n=1512)** | **16 years**  **(n=1663)** | **17 years**  **(n=1158)** | **18 years**  **(n=560)** |
| --- | --- | --- | --- | --- |
| Non-consumers of EDs | 41.9% (39.4–44.4) | 31.6% (29.4–33.9) | 30.7% (28.1–33.4) | 30.4% (26.5–34.2) |
| Occasional ED consumers | 34.9% (32.6–37.4) | 34.6% (32.3–36.9) | 36.8% (34.0–39.6) | 32.7% (28.8–36.6) |
| Moderate ED consumers | 14.3% (12.5–16.1) | 20.4% (18.5–22.4) | 18.6% (16.3–20.9) | 18.6% (15.3–21.8) |
| Regular ED consumers | 1.1% (0.6–1.7) | 1.6% (1.0–2.2) | 2.2% (1.4–3.1) | 2.0% (0.8–3.1) |
| Chronic low ED consumers | 3.1% (2.2–4.0) | 4.9% (3.9–6.0) | 5.7% (4.4–7.0) | 10.0% (7.5–12.5) |
| Chronic high ED consumers | 4.6% (3.6–5.7) | 6.7% (5.5–7.9) | 5.9% (4.6–7.3) | 6.4% (4.4–8.4) |

**Supplementary Table S7** 95% confidence intervals for the distribution of ED consumption groups within school type categories

|  | **Grammar school  (n=1449)** | **Secondary school  (n=2315)** | **Vocational school  (n=1129)** |
| --- | --- | --- | --- |
| Non-consumers of EDs | 44.4% (41.9–47.0) | 31.9% (30.0–33.8) | 26.8% (24.3–29.4) |
| Occasional ED consumers | 37.4% (34.9–39.9) | 34.9% (33.0–36.8) | 32.2% (29.5–35.0) |
| Moderate ED consumers | 11.8% (10.1–13.5) | 19.4% (17.8–21.1) | 22.5% (20.1–24.9) |
| Regular ED consumers | 0.5% (0.1–0.8) | 2.0% (1.4–2.6) | 2.4% (1.5–3.3) |
| Chronic low ED consumers | 3.2% (2.3–4.1) | 5.5% (4.6–6.5) | 6.9% (5.4–8.4) |
| Chronic high ED consumers | 2.7% (1.9–3.5) | 6.3% (5.3–7.3) | 9.1% (7.4–10.8) |

**Supplementary Table S8** 95% confidence intervals for the distribution of ED consumption groups across BMI percentile categories

|  | **Overall  population (n=4893)** | **Non-consumers of EDs  (n=1685)** | **Occasional  ED consumers  (n=1714)** | **Moderate  ED consumers  (n=875)** | **Regular  ED consumers  (n=80)** | **Chronic low  ED consumers  (n=252)** | **Chronic high  ED consumers  (n=287)** |
| --- | --- | --- | --- | --- | --- | --- | --- |
| Underweight (<P10) | 8.2% (7.4–8.9) | 10.9% (9.4–12.4) | 7.8% (6.5–9.0) | 5.4% (3.9–6.9) | 3.8% (0.0–7.9) | 4.0% (1.6–6.4) | 7.7% (4.6–10.7) |
| Normal weight (P10–P90) | 73.9% (72.6–75.1) | 76.0% (74.0–78.1) | 74.7% (72.7–76.8) | 68.9% (65.8–72.0) | 76.3% (66.9–85.6) | 69.8% (64.2–75.5) | 73.9% (68.8–79.0) |
| Overweight (P90–P97) | 9.9% (9.0–10.7) | 7.7% (6.4–9.0) | 8.9% (7.6–10.3) | 13.6% (11.3–15.9) | 11.3% (4.3–18.2) | 14.7% (10.3–19.1) | 12.2% (8.4–16.0) |
| Obesity (>P97) | 8.1% (7.3–8.9) | 5.3% (4.3–6.4) | 8.6% (7.3–9.9) | 12.1% (10.0–14.3) | 8.8% (2.6–14.9) | 11.5% (7.6–15.4) | 6.3% (3.5–9.1) |

**Supplementary Table S9** 95% confidence intervals for the distribution of ED consumption groups across smoking status categories

|  | **Non-consumers  of EDs  (n=1685)** | **Occasional  ED consumers  (n=1714)** | **Moderate  ED consumers  (n=875)** | **Regular  ED consumers  (n=80)** | **Chronic low  ED consumers  (n=252)** | **Chronic high  ED consumers  (n=287)** |
| --- | --- | --- | --- | --- | --- | --- |
| Smoker | 5.6% (4.5–6.7) | 17.6% (15.8–19.4) | 33.0% (29.9–36.1) | 37.5% (26.9–48.1) | 50.0% (43.8–56.2) | 53.3% (47.5–59.1) |
| Ex-smoker | 6.8% (5.6–8.0) | 16.1% (14.4–17.8) | 18.1% (15.5–20.6) | 17.5% (9.2–25.8) | 14.7% (10.3–19.1) | 18.5% (14.0–23.0) |
| Non-smoker | 85.8% (84.1–87.4) | 63.0% (60.7–65.3) | 45.5% (42.2–48.8) | 40.0% (29.3–50.7) | 32.5% (26.8–38.3) | 24.7% (19.7–29.7) |

Supplementary Table S10 Smoking behavior stratified by ED consumption groups

|  | **Overall population  (n=4893)** | **Non-consumers of EDs**  **(n=1685)** | **Occasional**  **ED consumers (n=1714)** | **Moderate**  **ED consumers (n=875)** | **Regular**  **ED consumers (n=80)** | **Chronic low**  **ED consumers (n=252)** | **Chronic high**  **ED consumers (n=287)** |
| --- | --- | --- | --- | --- | --- | --- | --- |
| **Smoker** | n=993 | n=94 | n=301 | n=289 | n=30 | n=126 | n=153 |
| **Smoking products** ^a^ | | | | | | | |
| Cigarettes | 63.6% (632) | 73.4% (69) | 62.1% (187) | 59.2% (171) | 56.7% (17) | 70.6% (89) | 64.7% (99) |
| E-cigarettes, e-shisha, tobacco heaters | 58.0% (576) | 35.1% (33) | 53.5% (161) | 62.3% (180) | 56.7% (17) | 55.6% (70) | 75.2% (115) |
| Shisha | 33.3% (331) | 17.0% (16) | 30.9% (93) | 31.8% (92) | 40.0% (12) | 35.7% (45) | 47.7% (73) |
| Don´t know/No answer | 0.7% (7) | 1.1% (1) | 1.0% (3) | 1.0% (3) | – | – | – |
| **Smoking cigarettes frequency** | | | | | | | |
| Less than once a week | 11.6% (73) | 14.5% (10) | 17.1% (32) | 7.6% (13) | 11.8% (2) | 10.1% (9) | 7.1% (7) |
| Once a week | 10.0% (63) | 20.3% (14) | 8.0% (15) | 11.7% (20) | 11.8% (2) | 10.1% (9) | 3.0% (3) |
| Multiple times a week | 18.7% (118) | 23.2% (16) | 23.0% (43) | 20.5% (35) | 5.9% (1) | 9.0% (8) | 15.2% (15) |
| Daily | 58.1% (367) | 40.6% (28) | 49.7% (93) | 57.9% (99) | 64.7% (11) | 69.7% (62) | 74.7% (74) |
| Don´t know/No answer | 1.7% (11) | 1.4% (1) | 2.1% (4) | 2.3% (4) | 5.9% (1) | 1.1% (1) | – |
| **Smoking cigarettes per day** ^b^ | 5.0 (1.1–10.0) | 2.0 (0.7–5.0) | 3.0 (0.7–6.5) | 5.0 (2.5–8.0) | 5.0 (1.1–10.0) | 7.0 (1.1–10.0) | 8.0 (4.0–12.5) |

Variables expressed as percentage (n) or median (IQR).

^a^ Multiple answers possible.

^b^ Missings: n=215.

Supplementary Table S11 Alcohol consumption in the last 12 month stratified by ED consumption groups

|  | **Overall population  (n=4893)** | **Non-consumers of EDs  (n=1685)** | **Occasional ED consumers (n=1714)** | **Moderate ED consumers (n=875)** | **Regular ED consumers (n=80)** | **Chronic low ED consumers (n=252)** | **Chronic high ED consumers (n=287)** | |
| --- | --- | --- | --- | --- | --- | --- | --- | --- |
| **Alcohol consumer** | n=3292 | n=918 | n=1243 | n=655 | n=56 | n=196 | n=224 | |
| **Alcohol frequency** | | | | | | | | |
| Once a month or less | 30.8% (1505) | 30.0% (505) | 35.3% (605) | 28.8% (252) | 21.3% (17) | 23.8% (60) | 23.0% (66) | |
| 2–3 times a month | 15.8% (773) | 9.4% (159) | 19.0% (326) | 18.6% (163) | 22.5% (18) | 20.6% (52) | 19.2% (55) | |
| Once a week | 7.0% (340) | 4.7% (79) | 7.1% (122) | 9.8% (86) | 7.5% (6) | 9.9% (25) | 7.7% (22) | |
| Twice a week | 5.5% (269) | 3.0% (51) | 4.3% (73) | 8.7% (76) | 5.0% (4) | 12.3% (31) | 11.9% (34) | |
| Three times a week | 1.8% (90) | 0.8% (14) | 1.3% (22) | 1.9% (17) | 6.3% (5) | 4.0% (10) | 7.7% (22) | |
| Four times a week | 0.8% (38) | 0.2% (4) | 0.6% (10) | 0.9% (8) | 2.5% (2) | 2.0% (5) | 3.1% (9) | |
| 5–6 times a week | 0.4% (19) | 0.1% (1) | 0.3% (5) | 0.3% (3) | – | 0.4% (1) | 3.1% (9) | |
| Daily | 0.3% (15) | 0.3% (5) | 0.1% (1) | 0.6% (5) | 1.3% (1) | – | 1.1% (3) | |
| Don´t know/No answer | 5.0% (243) | 5.9% (100) | 4.6% (79) | 5.1% (45) | 3.8% (3) | 4.8% (12) | 1.4% (4) | |
| **Alcohol quantity** **in one session** | | | | | | | |  |
| Half a glass | 10.1% (493) | 13.6% (229) | 9.7% (166) | 6.3% (55) | 8.8% (7) | 5.6% (14) | 7.7% (22) | |
| 1 glass | 12.4% (607) | 11.0% (186) | 14.3% (245) | 13.5% (118) | 6.3% (5) | 9.5% (24) | 10.1% (29) | |
| 2 glasses | 11.0% (536) | 7.7% (130) | 13.7% (234) | 12.1% (106) | 7.5% (6) | 11.1% (28) | 11.2% (32) | |
| 3 glasses | 8.9% (435) | 5.2% (88) | 10.3% (176) | 13.1% (115) | 8.8% (7) | 9.5% (24) | 8.7% (25) | |
| 4 glasses or more | 17.4% (852) | 7.8% (132) | 17.6% (302) | 23.0% (201) | 33.8% (27) | 33.3% (84) | 36.9% (106) | |
| Don´t know/No answer | 7.5% (369) | 9.1% (153) | 7.0% (120) | 6.9% (60) | 5.0% (4) | 8.7% (22) | 3.5% (10) | |

Supplementary Table S12 Leisure activities stratified by ED consumption groups

|  | **Overall population  (n=4893)** | **Non-consumers  of EDs  (n=1685)** | **Occasional ED consumers  (n=1714)** | **Moderate ED consumers  (n=875)** | **Regular ED consumers  (n=80)** | **Chronic low ED consumers  (n=252)** | **Chronic high ED consumers  (n=287)** |
| --- | --- | --- | --- | --- | --- | --- | --- |
| **Playing PlayStation, Xbox, Nintendo, computer games, etc.** | | | | | | | |
| Never | 25.6% (1252) | 29.7% (501) | 24.6% (422) | 23.1% (202) | 23.8% (19) | 20.6% (52) | 19.5% (56) |
| Less than once a week | 14.6% (714) | 15.0% (253) | 15.4% (264) | 13.9% (122) | 8.8% (7) | 13.5% (34) | 11.8% (34) |
| Once a week | 11.1% (542) | 10.1% (170) | 11.9% (204) | 11.2% (98) | 8.8% (7) | 13.5% (34) | 10.1% (29) |
| 2-6 times a week | 25.7% (1259) | 23.3% (393) | 26.3% (450) | 28.0% (245) | 25.0% (20) | 31.0% (78) | 25.4% (73) |
| Daily | 21.4% (1045) | 20.4% (343) | 20.0% (343) | 21.9% (192) | 30.0% (24) | 20.6% (52) | 31.7% (91) |
| Don´t know/No answer | 1.7% (81) | 1.5% (25) | 1.8% (31) | 1.8% (16) | 3.8% (3) | 0.8% (2) | 1.4% (4) |
| **Video calling with friends or other people (online via WhatsApp, FaceTime, Skype, etc.)** | | | | | | | |
| Never | 22.1% (1082) | 27.7% (467) | 21.2% (363) | 19.0% (166) | 18.8% (15) | 12.7% (32) | 13.6% (39) |
| Less than once a week | 20.4% (997) | 23.3% (392) | 19.9% (341) | 17.5% (153) | 18.8% (15) | 19.8% (50) | 16.0% (46) |
| Once a week | 14.6% (712) | 13.6% (229) | 14.8% (253) | 15.8% (138) | 13.8% (11) | 14.3% (36) | 15.7% (45) |
| 2-6 times a week | 22.7% (1109) | 20.0% (337) | 23.6% (404) | 25.4% (222) | 16.3% (13) | 27.0% (68) | 22.6% (65) |
| Daily | 18.0% (882) | 12.5% (211) | 18.6% (319) | 20.6% (180) | 31.3% (25) | 24.2% (61) | 30.0% (86) |
| Don´t know/No answer | 2.3% (111) | 2.9% (49) | 2.0% (34) | 1.8% (16) | 1.3% (1) | 2.0% (5) | 2.1% (6) |
| **Meeting friends/people outside of school hours** | | | | | | | |
| Never | 4.8% (237) | 7.3% (123) | 4.3% (74) | 3.0% (26) | 3.8% (3) | 1.2% (3) | 2.8% (8) |
| Less than once a week | 16.1% (787) | 23.8% (401) | 13.7% (234) | 10.1% (88) | 12.5% (10) | 8.3% (21) | 11.5% (33) |
| Once a week | 21.6% (1056) | 25.6% (432) | 21.3% (365) | 18.7% (164) | 20.0% (16) | 12.3% (31) | 16.7% (48) |
| 2-6 times a week | 45.3% (2217) | 35.5% (598) | 49.4% (847) | 53.6% (469) | 37.5% (30) | 58.7% (148) | 43.6% (125) |
| Daily | 9.9% (486) | 4.7% (80) | 9.2% (158) | 13.1% (115) | 22.5% (18) | 17.5% (44) | 24.7% (71) |
| Don´t know/No answer | 2.2% (110) | 3.0% (51) | 2.1% (36) | 1.5% (13) | 3.8% (3) | 2.0% (5) | 0.7% (2) |
| **Watching online videos on YouTube, TikTok, etc.** | | | | | | | |
| Never | 1.1% (56) | 1.7% (28) | 0.8% (13) | 1.0% (9) | 1.3% (1) | 0.8% (2) | 1.1% (3) |
| Less than once a week | 1.7% (83) | 2.7% (46) | 1.2% (20) | 1.0% (9) | 1.3% (1) | 0.8% (2) | 1.7% (5) |
| Once a week | 2.7% (134) | 3.8% (64) | 2.4% (41) | 2.1% (18) | 3.8% (3) | 1.6% (4) | 1.4% (4) |
| 2-6 times a week | 14.8% (726) | 19.5% (328) | 12.9% (221) | 12.8% (112) | 7.5% (6) | 9.5% (24) | 12.2% (35) |
| Daily | 79.0% (3867) | 71.6% (1206) | 82.4% (1412) | 82.6% (723) | 85.0% (68) | 86.5% (218) | 83.6% (240) |
| Don´t know/No answer | 0.6% (27) | 0.8% (13) | 0.4% (7) | 0.5% (4) | 1.3% (1) | 0.8% (2) | – |
| **Watching movies/series on streaming platforms (e.g., Netflix, Amazon Prime)** | | | | | | | |
| Never | 5.5% (268) | 9.2% (155) | 3.6% (61) | 3.8% (33) | 5.0% (4) | 2.8% (7) | 2.8% (8) |
| Less than once a week | 11.1% (541) | 14.1% (237) | 11.4% (195) | 8.3% (73) | 6.3% (5) | 4.0% (10) | 7.3% (21) |
| Once a week | 14.4% (704) | 15.6% (262) | 14.2% (244) | 14.3% (125) | 11.3% (9) | 10.3% (26) | 13.2% (38) |
| 2-6 times a week | 37.6% (1841) | 37.3% (629) | 39.0% (669) | 39.4% (345) | 38.8% (31) | 33.7% (85) | 28.6% (82) |
| Daily | 30.4% (1488) | 22.7% (383) | 30.8% (527) | 33.4% (292) | 37.5% (30) | 47.6% (120) | 47.4% (136) |
| Don´t know/No answer | 1.0% (51) | 1.1% (19) | 1.1% (18) | 0.8% (7) | 1.3% (1) | 1.6% (4) | 0.7% (2) |
| **Watching television, DVDs** | | | | | | | |
| Never | 39.8% (1948) | 35.6% (599) | 40.5% (694) | 42.7% (374) | 41.3% (33) | 46.8% (118) | 45.3% (130) |
| Less than once a week | 24.6% (1202) | 25.6% (431) | 25.9% (444) | 23.0% (201) | 21.3% (17) | 21.4% (54) | 19.2% (55) |
| Once a week | 13.4% (656) | 15.1% (255) | 12.7% (218) | 12.3% (108) | 12.5% (10) | 10.3% (26) | 13.6% (39) |
| 2-6 times a week | 12.6% (615) | 13.9% (234) | 12.2% (209) | 12.7% (111) | 13.8% (11) | 9.9% (25) | 8.7% (25) |
| Daily | 7.4% (362) | 7.5% (127) | 6.6% (113) | 6.6% (58) | 10.0% (8) | 8.3% (21) | 12.2% (35) |
| Don´t know/No answer | 2.3% (110) | 2.3% (39) | 2.1% (36) | 2.6% (23) | 1.3% (1) | 3.2% (8) | 1.1% (3) |
| **Reading (books, eBooks, magazines, etc.)** | | | | | | | |
| Never | 29.1% (1422) | 19.0% (320) | 29.7% (509) | 37.4% (327) | 46.3% (37) | 43.7% (110) | 41.5% (119) |
| Less than once a week | 19.8% (970) | 19.2% (323) | 22.1% (379) | 19.7% (172) | 16.3% (13) | 15.9% (40) | 15.0% (43) |
| Once a week | 16.6% (811) | 17.4% (294) | 16.9% (290) | 16.5% (144) | 16.3% (13) | 12.7% (32) | 13.2% (38) |
| 2-6 times a week | 19.7% (964) | 24.5% (413) | 18.0% (309) | 16.3% (143) | 11.3% (9) | 13.5% (34) | 19.5% (56) |
| Daily | 12.8% (628) | 18.0% (304) | 11.0% (188) | 8.7% (76) | 6.3% (5) | 11.9% (30) | 8.7% (25) |
| Don´t know/No answer | 2.0% (98) | 1.8% (31) | 2.3% (39) | 1.5% (13) | 3.8% (3) | 2.4% (6) | 2.1% (6) |
| **Reading/listening to the news** | | | | | | | |
| Never | 13.8% (676) | 11.3% (190) | 13.7% (234) | 15.3% (134) | 23.8% (19) | 17.1% (43) | 19.5% (56) |
| Less than once a week | 14.8% (723) | 14.0% (236) | 15.8% (271) | 15.0% (131) | 15.0% (12) | 14.3% (36) | 12.9% (37) |
| Once a week | 15.2% (743) | 14.0% (235) | 15.9% (273) | 15.4% (135) | 16.3% (13) | 13.9% (35) | 18.1% (52) |
| 2-6 times a week | 21.4% (1045) | 24.2% (408) | 21.0% (360) | 20.0% (175) | 20.0% (16) | 19.8% (50) | 12.5% (36) |
| Daily | 32.7% (1601) | 34.1% (574) | 31.2% (534) | 32.6% (285) | 25.0% (20) | 34.1% (86) | 35.5% (102) |
| Don´t know/No answer | 2.2% (105) | 2.5% (42) | 2.5% (42) | 1.7% (15) | – | 0.8% (2) | 1.4% (4) |
| **Doing something with the family** | | | | | | | |
| Never | 4.3% (210) | 4.0% (67) | 3.4% (58) | 3.9% (34) | 16.3% (13) | 6.4% (16) | 7.7% (22) |
| Less than once a week | 18.5% (904) | 17.3% (291) | 19.3% (331) | 16.9% (148) | 20.0% (16) | 20.6% (52) | 23.0% (66) |
| Once a week | 32.4% (1587) | 32.3% (544) | 33.4% (573) | 33.4% (292) | 25.0% (20) | 32.1% (81) | 26.8% (77) |
| 2-6 times a week | 29.9% (1464) | 30.5% (514) | 30.4% (521) | 31.0% (271) | 16.3% (13) | 26.6% (67) | 27.2% (78) |
| Daily | 11.4% (559) | 12.9% (218) | 10.3% (176) | 11.0% (96) | 16.3% (13) | 10.7% (27) | 10.1% (29) |
| Don´t know/No answer | 3.5% (169) | 3.0% (51) | 3.2% (55) | 3.9% (34) | 6.3% (5) | 3.6% (9) | 5.2% (15) |
| **Engaging in a club, project, or initiative** | | | | | | | |
| Never | 53.4% (2611) | 48.2% (812) | 55.2% (946) | 54.7% (479) | 53.8% (43) | 63.1% (159) | 59.9% (172) |
| Less than once a week | 10.5% (513) | 10.8% (182) | 10.9% (186) | 9.7% (85) | 8.8% (7) | 11.1% (28) | 8.7% (25) |
| Once a week | 9.9% (485) | 12.2% (205) | 9.2% (157) | 8.2% (72) | 11.3% (9) | 6.8% (17) | 8.7% (25) |
| 2-6 times a week | 14.7% (717) | 15.8% (266) | 13.9% (238) | 16.0% (140) | 13.8% (11) | 10.3% (26) | 12.5% (36) |
| Daily | 3.5% (170) | 4.2% (70) | 3.1% (53) | 2.7% (24) | 6.3% (5) | 2.4% (6) | 4.2% (12) |
| Don´t know/No answer | 8.1% (397) | 8.9% (150) | 7.8% (134) | 8.6% (75) | 6.3% (5) | 6.4% (16) | 5.9% (17) |
| **Doing something creative, artistic** | | | | | | | |
| Never | 29.1% (1423) | 23.3% (392) | 28.7% (491) | 34.5% (302) | 46.3% (37) | 36.9% (93) | 37.6% (108) |
| Less than once a week | 22.0% (1075) | 22.1% (372) | 23.3% (400) | 20.6% (180) | 21.3% (17) | 21.4% (54) | 18.1% (52) |
| Once a week | 18.8% (921) | 20.2% (340) | 19.3% (331) | 18.7% (164) | 11.3% (9) | 14.7% (37) | 13.9% (40) |
| 2-6 times a week | 18.1% (884) | 20.8% (350) | 16.9% (290) | 15.5% (136) | 13.8% (11) | 16.3% (41) | 19.5% (56) |
| Daily | 7.9% (386) | 9.8% (165) | 6.9% (118) | 6.7% (59) | 6.3% (5) | 6.4% (16) | 8.0% (23) |
| Don´t know/No answer | 4.2% (204) | 3.9% (66) | 4.9% (84) | 3.9% (34) | 1.3% (1) | 4.4% (11) | 2.8% (8) |
| **Playing an instrument, making music or singing (e.g., in a choir)** | | | | | | | |
| Never | 62.1% (3037) | 55.5% (935) | 62.3% (1067) | 68.6% (600) | 70.0% (56) | 70.6% (178) | 70.0% (201) |
| Less than once a week | 8.5% (416) | 9.1% (153) | 9.6% (165) | 7.0% (61) | 6.3% (5) | 7.1% (18) | 4.9% (14) |
| Once a week | 9.5% (465) | 10.1% (170) | 10.0% (172) | 9.0% (79) | 7.5% (6) | 6.0% (15) | 8.0% (23) |
| 2-6 times a week | 10.9% (533) | 14.5% (244) | 9.6% (165) | 7.7% (67) | 8.8% (7) | 10.7% (27) | 8.0% (23) |
| Daily | 7.0% (342) | 8.2% (139) | 6.8% (116) | 5.7% (50) | 6.3% (5) | 3.6% (9) | 8.0% (23) |
| Don´t know/No answer | 2.0% (100) | 2.6% (44) | 1.7% (29) | 2.1% (18) | 1.3% (1) | 2.0% (5) | 1.0% (3) |
| **Going shopping** | | | | | | | |
| Never | 16.3% (797) | 22.3% (375) | 15.1% (259) | 9.6% (84) | 12.5% (10) | 12.3% (31) | 13.2% (38) |
| Less than once a week | 51.0% (2495) | 53.2% (896) | 52.3% (897) | 50.1% (438) | 41.3% (33) | 44.1% (111) | 41.8% (120) |
| Once a week | 17.5% (856) | 13.3% (224) | 18.1% (311) | 21.1% (185) | 22.5% (18) | 21.4% (54) | 22.3% (64) |
| 2-6 times a week | 7.6% (372) | 5.1% (86) | 7.2% (123) | 9.7% (85) | 11.3% (9) | 11.5% (29) | 13.9% (40) |
| Daily | 1.8% (88) | 1.3% (22) | 1.3% (23) | 2.1% (18) | 2.5% (2) | 4.0% (10) | 4.5% (13) |
| Don´t know/No answer | 5.8% (285) | 4.9% (82) | 5.9% (101) | 7.4% (65) | 10.0% (8) | 6.8% (17) | 4.2% (12) |
| **Shopping online** | | | | | | | |
| Never | 26.4% (1293) | 33.9% (571) | 24.9% (426) | 20.1% (176) | 30.0% (24) | 19.1% (48) | 16.7% (48) |
| Less than once a week | 44.8% (2192) | 46.4% (781) | 47.0% (805) | 44.2% (387) | 31.3% (25) | 34.1% (86) | 37.6% (108) |
| Once a week | 14.5% (709) | 10.2% (172) | 14.5% (248) | 18.1% (158) | 20.0% (16) | 20.2% (51) | 22.3% (64) |
| 2-6 times a week | 6.9% (338) | 3.3% (56) | 6.9% (118) | 8.8% (77) | 8.8% (7) | 17.1% (43) | 12.9% (37) |
| Daily | 2.2% (107) | 1.3% (22) | 1.7% (29) | 2.3% (20) | 3.8% (3) | 4.0% (10) | 8.0% (23) |
| Don´t know/No answer | 5.2% (254) | 4.9% (83) | 5.1% (88) | 6.5% (57) | 6.3% (5) | 5.6% (14) | 2.4% (7) |

Supplementary Table S13 Daily duration of different types of media use stratified by ED consumption groups

|  | **Overall population  (n=4893)** | **Non-consumers of EDs  (n=1685)** | **Occasional ED consumers (n=1714)** | **Moderate ED consumers (n=875)** | **Regular ED consumers (n=80)** | **Chronic low-ED consumers (n=252)** | **Chronic high-ED consumers (n=287)** |
| --- | --- | --- | --- | --- | --- | --- | --- |
| **Gaming on console, computer, tablet, smartphone, etc.** | | | | | | | |
| Not at all | 24.2% (1185) | 28.6% (482) | 23.4% (401) | 21.3% (186) | 20.0% (16) | 19.4% (49) | 17.8% (51) |
| Up to 1 hour | 23.7% (1162) | 23.7% (399) | 25.5% (437) | 23.7% (207) | 15.0% (12) | 24.6% (62) | 15.7% (45) |
| Up to 3 hours | 23.8% (1163) | 24.7% (417) | 23.9% (410) | 22.3% (195) | 23.8% (19) | 24.2% (61) | 21.3% (61) |
| More than 3 hours | 25.9% (1265) | 20.8% (350) | 24.4% (418) | 30.3% (265) | 40.0% (32) | 29.8% (75) | 43.6% (125) |
| Don´t know/No answer | 2.4% (118) | 2.2% (37) | 2.8% (48) | 2.5% (22) | 1.3% (1) | 2.0% (5) | 1.7% (5) |
| **Using messengers like (WhatsApp, Signal, Telegram on smartphone, tablet, etc.)** | | | | | | | |
| Not at all | 2.0% (96) | 3.6% (61) | 1.5% (25) | 0.8% (7) | 1.3% (1) | 0.4% (1) | 0.3% (1) |
| Up to 1 hour | 53.7% (2626) | 64.9% (1094) | 53.7% (920) | 45.4% (397) | 38.8% (31) | 39.7% (100) | 29.3% (84) |
| Up to 3 hours | 25.1% (1230) | 19.7% (332) | 26.3% (450) | 29.6% (259) | 21.3% (17) | 30.6% (77) | 33.1% (95) |
| More than 3 hours | 16.8% (821) | 8.7% (147) | 16.0% (274) | 22.4% (196) | 37.5% (30) | 28.2% (71) | 35.9% (103) |
| Don´t know/No answer | 2.5% (120) | 3.0% (51) | 2.6% (45) | 1.8% (16) | 1.3% (1) | 1.2% (3) | 1.4% (4) |
| **Using social networks (Facebook, Instagram, Twitter on smartphone, tablet, etc.)** | | | | | | | |
| Not at all | 11.6% (567) | 22.0% (371) | 7.4% (127) | 4.6% (40) | 8.8% (7) | 3.2% (8) | 4.9% (14) |
| Up to 1 hour | 37.2% (1819) | 39.1% (658) | 37.9% (650) | 36.0% (315) | 37.5% (30) | 31.3% (79) | 30.3% (87) |
| Up to 3 hours | 29.4% (1438) | 25.9% (437) | 32.2% (552) | 31.4% (275) | 22.5% (18) | 30.6% (77) | 27.5% (79) |
| More than 3 hours | 20.2% (987) | 11.0% (185) | 20.6% (353) | 26.6% (233) | 30.0% (24) | 34.5% (87) | 36.6% (105) |
| Don´t know/No answer | 1.7% (82) | 2.0% (34) | 1.9% (32) | 1.4% (12) | 1.3% (1) | 0.4% (1) | 0.7% (2) |
| **Watching online videos, using streaming platforms** | | | | | | | |
| Not at all | 9.0% (441) | 9.7% (164) | 7.9% (136) | 8.8% (77) | 10.0% (8) | 9.1% (23) | 11.5% (33) |
| Up to 1 hour | 22.5% (1100) | 26.7% (450) | 21.6% (370) | 21.0% (184) | 17.5% (14) | 16.7% (42) | 13.9% (40) |
| Up to 3 hours | 38.9% (1905) | 39.4% (664) | 41.4% (710) | 37.4% (327) | 33.8% (27) | 33.3% (84) | 32.4% (93) |
| More than 3 hours | 27.1% (1328) | 21.3% (358) | 27.0% (462) | 30.3% (265) | 33.8% (27) | 38.9% (98) | 41.1% (118) |
| Don´t know/No answer | 2.4% (119) | 2.9% (49) | 2.1% (36) | 2.5% (22) | 5.0% (4) | 2.0% (5) | 1.1% (3) |
| **Watching television, DVDs** | | | | | | | |
| Not at all | 55.1% (2697) | 51.7% (871) | 57.1% (979) | 56.3% (493) | 50.0% (40) | 60.7% (153) | 56.1% (161) |
| Up to 1 hour | 28.1% (1377) | 31.0% (522) | 27.3% (467) | 26.3% (230) | 32.5% (26) | 23.8% (60) | 25.1% (72) |
| Up to 3 hours | 10.1% (494) | 10.7% (180) | 9.6% (165) | 10.2% (89) | 7.5% (6) | 9.9% (25) | 10.1% (29) |
| More than 3 hours | 2.9% (144) | 2.5% (42) | 2.6% (44) | 3.4% (30) | 5.0% (4) | 3.2% (8) | 5.6% (16) |
| Don´t know/No answer | 3.7% (181) | 4.2% (70) | 3.4% (59) | 3.8% (33) | 5.0% (4) | 2.4% (6) | 3.1% (9) |
| **Reading (books, eBooks, magazines, newspapers, comics, etc.)** | | | | | | | |
| Not at all | 36.4% (1782) | 25.9% (437) | 37.7% (647) | 45.5% (398) | 47.5% (38) | 50.8% (128) | 46.7% (134) |
| Up to 1 hour | 39.6% (1936) | 44.4% (748) | 39.7% (681) | 35.0% (306) | 32.5% (26) | 32.1% (81) | 32.8% (94) |
| Up to 3 hours | 13.1% (641) | 16.0% (270) | 12.4% (213) | 11.0% (96) | 11.3% (9) | 10.3% (26) | 9.4% (27) |
| More than 3 hours | 6.7% (326) | 9.0% (152) | 5.7% (97) | 4.6% (40) | 3.8% (3) | 4.4% (11) | 8.0% (23) |
| Don´t know/No answer | 4.3% (208) | 4.6% (78) | 4.4% (76) | 4.0% (35) | 5.0% (4) | 2.4% (6) | 3.1% (9) |
| **Listening to music** | | | | | | | |
| Not at all | 3.4% (168) | 5.5% (93) | 2.9% (50) | 1.7% (15) | 2.5% (2) | 0.8% (2) | 2.1% (6) |
| Up to 1 hour | 22.9% (1121) | 27.8% (469) | 22.5% (386) | 21.4% (187) | 8.8% (7) | 13.5% (34) | 13.2% (38) |
| Up to 3 hours | 30.9% (1510) | 31.2% (526) | 32.4% (556) | 30.9% (270) | 31.3% (25) | 29.4% (74) | 20.6% (59) |
| More than 3 hours | 41.6% (2037) | 33.5% (565) | 41.4% (709) | 45.0% (394) | 56.3% (45) | 56.0% (141) | 63.8% (183) |
| Don´t know/No answer | 1.2% (57) | 1.9% (32) | 0.8% (13) | 1.0% (9) | 1.3% (1) | 0.4% (1) | 0.3% (1) |
| **Surfing the internet** | | | | | | | |
| Not at all | 7.0% (340) | 9.2% (155) | 6.8% (116) | 4.1% (36) | 5.0% (4) | 8.3% (21) | 2.8% (8) |
| Up to 1 hour | 45.2% (2209) | 49.4% (832) | 46.3% (793) | 43.0% (376) | 26.3% (21) | 37.3% (94) | 32.4% (93) |
| Up to 3 hours | 19.1% (936) | 17.3% (292) | 19.9% (341) | 19.3% (169) | 25.0% (20) | 19.4% (49) | 22.7% (65) |
| More than 3 hours | 24.1% (1180) | 18.1% (305) | 22.6% (387) | 29.6% (259) | 42.5% (34) | 32.1% (81) | 39.7% (114) |
| Don´t know/No answer | 4.7% (228) | 6.0% (101) | 4.5% (77) | 4.0% (35) | 1.3% (1) | 2.8% (7) | 2.4% (7) |

**Supplementary Table S14** 95% confidence intervals for the distribution of ED consumption groups across screentime categories

|  | **Non-consumers  of EDs  (n=1685)** | **Occasional  ED consumers  (n=1714)** | **Moderate  ED consumers  (n=875)** | **Regular  ED consumers  (n=80)** | **Chronic low  ED consumers  (n=252)** | **Chronic high  ED consumers  (n=287)** |
| --- | --- | --- | --- | --- | --- | --- |
| Screentime up to 5 h/day | 29.0% (26.8–31.1) | 18.7% (16.9–20.6) | 15.7% (13.2–18.1) | 12.5% (5.3–19.7) | 14.3% (10.0–18.6) | 5.6% (2.9–8.2) |
| Screentime up to 8 h/day | 30.9% (28.7–33.1) | 29.6% (27.4–31.7) | 24.2% (21.4–27.1) | 18.8% (10.2–27.3) | 22.6% (17.5–27.8) | 20.2% (15.6–24.9) |
| Screentime more than 8 h/day | 35.8% (33.5–38.1) | 48.5% (46.2–50.9) | 57.7% (54.4–61.0) | 65.0% (54.5–75.5) | 61.9% (55.9–67.9) | 72.8% (67.7–77.9) |
